# Skeletal Muscles of Sedentary and Physically Active Aged People Have Distinctive Genic Extrachromosomal Circular DNA Profiles

**DOI:** 10.3390/ijms24032736

**Published:** 2023-02-01

**Authors:** Daniela Gerovska, Marcos J. Araúzo-Bravo

**Affiliations:** 1 Computational Biology and Systems Biomedicine, Biodonostia Health Research Institute, Calle Doctor Begiristain s/n, 20014 San Sebastian, Spain; 2 Basque Foundation for Science, IKERBASQUE, Calle María Díaz Harokoa 3, 48013 Bilbao, Spain; 3 CIBER of Frailty and Healthy Aging (CIBERfes), 28029 Madrid, Spain; 4 Max Planck Institute for Molecular Biomedicine, Computational Biology and Bioinformatics, Röntgenstr. 20, 48149 Münster, Germany; 5 Department of Cell Biology and Histology, Faculty of Medicine and Nursing, University of Basque Country (UPV/EHU), 48940 Leioa, Spain

**Keywords:** extrachromosomal, eccDNA, skeletal muscle, active, sedentary, exercise, differential, circular DNA, aging, sarcopenia

## Abstract

To bring new extrachromosomal circular DNA (eccDNA) enrichment technologies closer to the clinic, specifically for screening, early diagnosis, and monitoring of diseases or lifestyle conditions, it is paramount to identify the differential pattern of the genic eccDNA signal between two states. Current studies using short-read sequenced purified eccDNA data are based on absolute numbers of unique eccDNAs per sample or per gene, length distributions, or standard methods for RNA-seq differential analysis. Previous analyses of RNA-seq data found significant transcriptomics difference between sedentary and active life style skeletal muscle (SkM) in young people but very few in old. The first attempt using circulomics data from SkM and blood of aged lifelong sedentary and physically active males found no difference at eccDNA level. To improve the capability of finding differences between circulomics data groups, we designed a computational method to identify Differentially Produced per Gene Circles (DPpGCs) from short-read sequenced purified eccDNA data based on the circular junction, split-read signal, of the eccDNA, and implemented it into a software tool DifCir in Matlab. We employed DifCir to find to the distinctive features of the influence of the physical activity or inactivity in the aged SkM that would have remained undetected by transcriptomics methods. We mapped the data from tissue from SkM and blood from two groups of aged lifelong sedentary and physically active males using Circle_finder and subsequent merging and filtering, to find the number and length distribution of the unique eccDNA. Next, we used DifCir to find up-DPpGCs in the SkM of the sedentary and active groups. We assessed the functional enrichment of the DPpGCs using Disease Gene Network and Gene Set Enrichment Analysis. To find genes that produce eccDNA in a group without comparison with another group, we introduced a method to find Common PpGCs (CPpGCs) and used it to find CPpGCs in the SkM of the sedentary and active group. Finally, we found the eccDNA that carries whole genes. We discovered that the eccDNA in the SkM of the sedentary group is not statistically different from that of physically active aged men in terms of number and length distribution of eccDNA. In contrast, with DifCir we found distinctive gene-associated eccDNA fingerprints. We identified statistically significant up-DPpGCs in the two groups, with the top up-DPpGCs shed by the genes *AGBL4*, *RNF213*, *DNAH7*, *MED13*, and *WWTR1* in the sedentary group, and *ZBTB7C*, *TBCD*, *ITPR2*, and *DDX11-AS1* in the active group. The up-DPpGCs in both groups carry mostly gene fragments rather than whole genes. Though the subtle transcriptomics difference, we found *RYR1* to be both transcriptionally up-regulated and up-DPpGCs gene in sedentary SkM. DifCir emphasizes the high sensitivity of the circulome compared to the transcriptome to detect the molecular fingerprints of exercise in aged SkM. It allows efficient identification of gene hotspots that excise more eccDNA in a health state or disease compared to a control condition.

## 1. Introduction

Chromosome-derived extrachromosomal circular DNA elements (eccDNA) of size ranging from tens to hundreds of thousands of base pairs have been detected in all eukaryotes examined so far [1], in normal human tissues [2,3], in cancer [4], and in plasma and serum [5,6]. A large amount of long, oncogenic extrachromosomal DNA elements (ecDNA) has been detected in human tumors with major oncogene transcripts directly derived from ecDNA [7,8]. The ecDNA chromatin is highly open and can express large amounts of oncogenes; it lacks centromeres and cannot follow Mendelian laws of inheritance, making ecDNA an important mechanism for driving tumor heterogeneity [9,10]. EccDNA purification protocols are sequences of steps to enrich and amplify eccDNA to improve its detection rate, followed by high throughput Whole Genome Sequencing (WGS) and bioinformatics analysis for eccDNA identification. EccDNA purification protocols, Circle-Seq [11] being one of them, are constantly being improved [9,12], and the number of tools for detection of eccDNA from short- (Circle-Map [13], Circle_finder [14], ecc_finder [15], eccSplorer [16]), and long-read WGS (CIDER-seq Data Analysis Software 2 [17], ecc_finder [15]) is fast growing.

To bring new technologies closer to the clinic, specifically for screening, early diagnosis, and monitoring of cancer and other disorders, it is important to identify the differential pattern of gene origin of the eccDNA signal between two conditions, such as disease progression, treatment response, or lifestyle choice. Currently, this is limited to counting and comparison of the absolute numbers of unique eccDNA per sample or per gene, comparison of length distributions, or standard methods for RNA-seq data analysis [18], when using short-read sequenced purified eccDNA data. However, the use of different eccDNA mapping methods, and/or the use of the same eccDNA purification protocol on a sample in different labs, does not result in the same unique eccDNA sequences and count [13,16]. Therefore, we designed a robust method to identify differentially produced per gene eccDNA (DPpGCs) that can carry exons, or mostly fragments of the gene, from purified eccDNA data based on the split-read signal of the eccDNA. Thus, we can identify genes or hotspots in the chromosomal DNA that produce eccDNA distinctly under two conditions.

Regular physical activity is beneficial for older adults with stronger muscles helping reduce the risk of falling and improving the ability to perform the routine tasks of daily life. The response of the skeletal muscle (SkM) to exercise changes with aging, with a shift from a predominantly anabolic response to limited gain of muscle strength and endurance [19]. Exercise training enables the SkM to counteract age-related sarcopenia by inducing a wide range of adaptations, sustained by the expression of protein-coding genes involved in energy handling, proteostasis, cytoskeletal organization, inflammation control, and cellular senescence [20]. While transcriptome studies on SkM adaptations to the exercise of young healthy subjects have identified upregulation in a number of signaling pathways following exercise, indicating enhanced mitochondrial biogenesis and mitochondrial oxidative responses, transcriptional response to exercise training was found to be weaker in elderly patients [19]. The physiological role of eccDNA in the SkM is still unknown. Understanding the differences between the eccDNA profiles of aged people who have been active and exercised regularly through their lifetime and people who have led lifelong sedentary lifestyle can have clinical interest for understanding and optimizing exercise-based therapies for sarcopenia.

The aim of this study was two-fold, namely, to introduce a new computational method for differential analysis of whole genome sequenced purified circular DNA data, and to characterize the circular DNA profiles and differences induced by lifelong exercise activity. Here, we proved the effectiveness of the DifCir method on short-read sequenced purified eccDNA Circle-Seq data from the SkM of two groups of healthy aged men, who had either performed regular exercise or remained sedentary for their entire lives (average age 62 years) [3,21], and whose previous analysis had not reported any difference between the two groups [3].

## 2. Results

### 2.1. Aim, Design, and Setting of the Study

**Figure 1:**
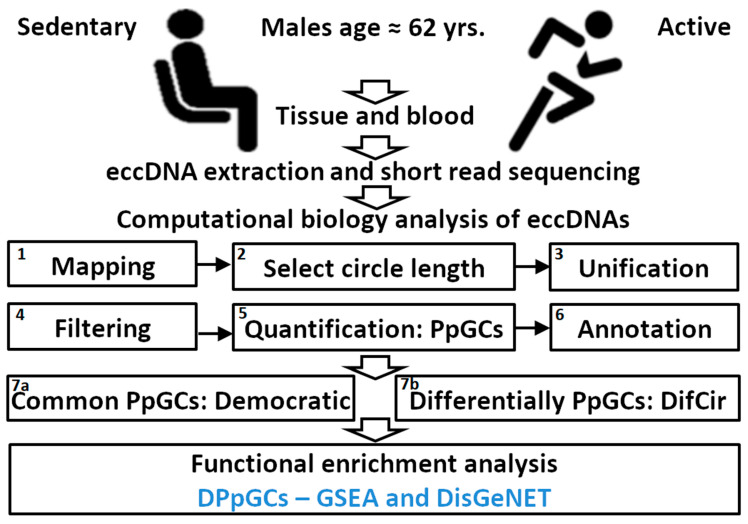
Experimental setup and computational analysis workflow. Isolation and purification of circular DNA from skeletal muscle (SkM) tissue (T) and blood (B) of sedentary (S) and active (A) individuals and subsequent assembly, annotation, quantification of eccDNA species, quantification of produced per gene circles (PpGCs), calculation of differentially PpGCs (DPpGCs), and identification of common PpGCs (CPpGCs) in the TS and TA groups using a democratic vote method. Functional enrichment analysis of the DPpGCs performed with GSEA and DisGeNET.

The following aims were instigated: Develop a new computational method and tool for identifying genomic regions that produce differentially eccDNA from gene fragments in two different states like disease versus control, degrees of illness development, response to treatment, sports activity; demonstrate the method on a short-read sequenced circulomics data set of human SkM from active and sedentary participants of similar age and same sex, and characterize the eccDNA profiles and differences induced by lifelong exercise activity. The experimental setup and computational analysis workflow are presented in Figure 1.

### 2.2. Numbers and Distributions of Unique eccDNA Are Similar in Sedentary and Active Aged SkM

**Figure 2:**
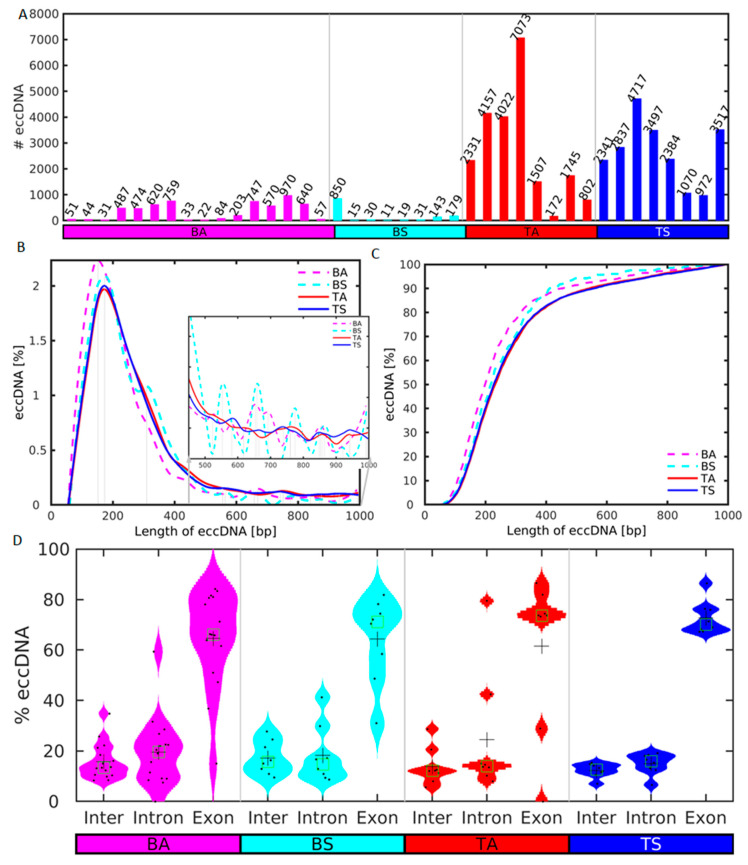
Distributions of number of unique sequence and length of eccDNA in sedentary (S) and active (A) men in SkM tissue (T) and blood (B). (**A**) Number of unique eccDNAs in each sample of the S and A groups up to a size of 10^4^ bp after merging and removal of eccDNA with less than 2 split reads. (**B**) Periodic enrichment of eccDNAs in the two groups in the size range from 0 to 10^3^ bp. The vertical lines mark the local maxima of the more abundant lengths after smoothing. (**C**) Cumulative distribution of the lengths of the eccDNAs in the range from 0 to 10^3^ bp. The S and A samples are depicted in blue and red, respectively. (**D**) Violin plots of the distribution of the length of the sequences of the eccDNAs in intergenic, and intron and exon genic regions. Data points are plotted with black dots, mean and median are shown as crosses and squares, respectively.

We first checked for possible differences in the number and length frequency distributions of the eccDNA in the blood and skeletal muscle (SkM) of the sedentary and active groups. The cross-chromosomal load of unique eccDNA up to a size of 100,000 bp ranges from 972 to 4717 unique eccDNA (mean ± sem 2726 ± 796) in sedentary (TS), and 172 to 7073 eccDNA (mean ± sem 2667 ± 448) in active (TA) SkM (Figure 2A). Though the eccDNA mean of TS is higher than the TA mean, the Wilcoxon rank sum test does not indicate that the unique eccDNA number in TA is statistically significantly greater than in TS at the 1% significance level (*p*-value = 0.677). Thus, the 1.0-fold increase in the number of eccDNA in TA compared to TS is not statistically significant.

The eccDNA number in active blood samples (BA, mean ± sem 158 ± 75) is very similar to that in sedentary blood (BS, mean ± sem 160 ± 101). However, the Wilcoxon rank sum test indicates that the eccDNA number of TS is statistically significantly higher (16.7-fold) than in BS (*p*-value = 7.77^−5^). Additionally, the eccDNA number in TA is statistically significantly higher (16.7-fold) than in BA (*p*-value= 3.68^−4^). Circular DNA exhibits enrichment for a length of around 160 bp for both groups (Figure 2B). This enrichment length is similar to the nucleosome repeat length (NRL), ~147 bp nucleosome length plus linker DNA. Such enrichment for multiples of this length is observed for the plasma samples but not for the SkM. The cumulative distribution of the lengths of the eccDNA shows that the majority of the eccDNA is shorter than 1000 bp (Figure 2C). Thus, neither the number of unique eccDNA nor their length frequencies differentiate between the sedentary and aged groups.

We calculated the distribution of the lengths of the sequences of the eccDNAs across intergenic and genic regions, as well as the eccDNA specifically excised from introns and exons (Figure 2D). For all analyzed samples the lengths of the eccDNAs in exonic regions are significantly longer than in intronic and intergenic regions using the Wilcoxon rank sum test at the 1% significance level.

Skeletal muscle mitochondria are implicated with age-related loss of function and insulin resistance. An assessment of the impact of physical activity upon the SkM mitochondria in elderly men and women (age 67.3 ± 0.6 years) showed robust improvement in SkM mitochondrial content and function in elderly people in response to a program of physical activity of moderate intensity [22]. Next, we asked whether mitochondrial DNA (MtDNA) is more in TA compared to TS aged SkM. We checked the number of the MtDNA in the blood and SkM samples (Figure S1). Most of the known pipelines (Circle-Map, ecc_finder), including our approach for mapping and counting unique eccDNA sequences, merge the sequences with overlaps and these merged sequences are consequently counted as unique eccDNA numbers. This approach results in mapping of one single unique MtDNA with the full length of the mitochondrial DNA of approximately 16 Kb. Additionally, the eccDNA purification methods do not seem to amplify homogeneously all parts of the genome. Therefore, in an attempt to quantify the MtDNA “copy number” content in each sample, we used the number of split reads corresponding to the MtDNA per sample obtained directly with Circle_finder. Thus, we counted the MtDNA with both fragments and the complete MtDNA sequence. Almost no MtDNA were detected in the plasma samples. The mean number of MtDNA in TA (mean ± sem 1511 ± 672) is 0.56-fold higher compared to TS (mean ± sem 945 ± 203). However, the difference is not statistically significant (*p*-value = 0.561) and the results are inconclusive.

### 2.3. Differential Analysis Based on Split-Reads Identifies Distinctive Genic eccDNA Profiles in Sedentary and Active SkM

**Figure 3:**
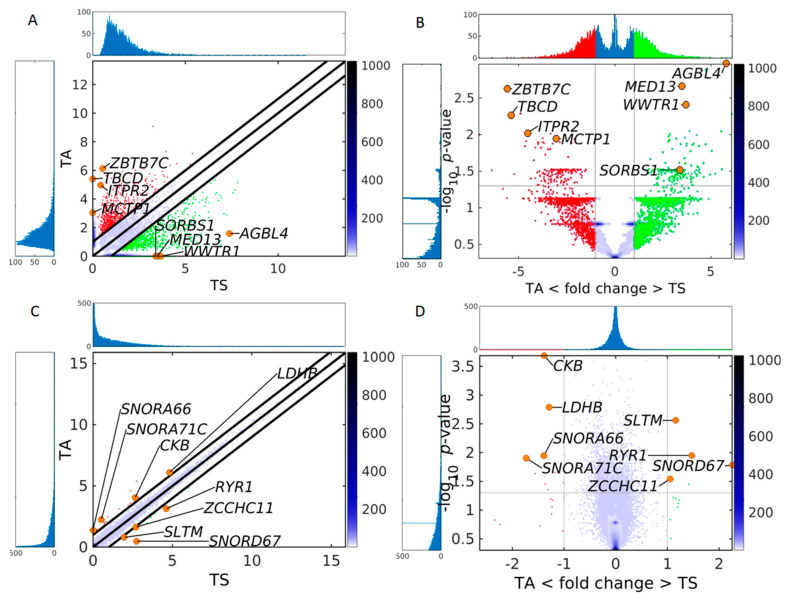
Comparison of the sensitivity of circulomics and transcriptomics data from SkM to detect differences between sedentary and active individuals. (**A**) Pairwise scatter plot and (**B**) volcano plot of circulomics data from tissue sedentary (TS) and tissue active (TA). (**C**) Pairwise scatter plot and (**D**) volcano plot of RNA-seq data from TS and TA. In all plots the color bar indicates the scattering density. Darker blue color corresponds to higher scattering density. In the scatter plot the up-DPpGCs in the TA samples (ordinate) are shown with red dots, and up-DPpGCs and DEGs in the TS samples (abscissa), with green. Several gene positions are shown as orange circles. The levels are log_2_-scaled. The histograms visualize the eccDNA production and gene expression spectra.

**Figure 4:**
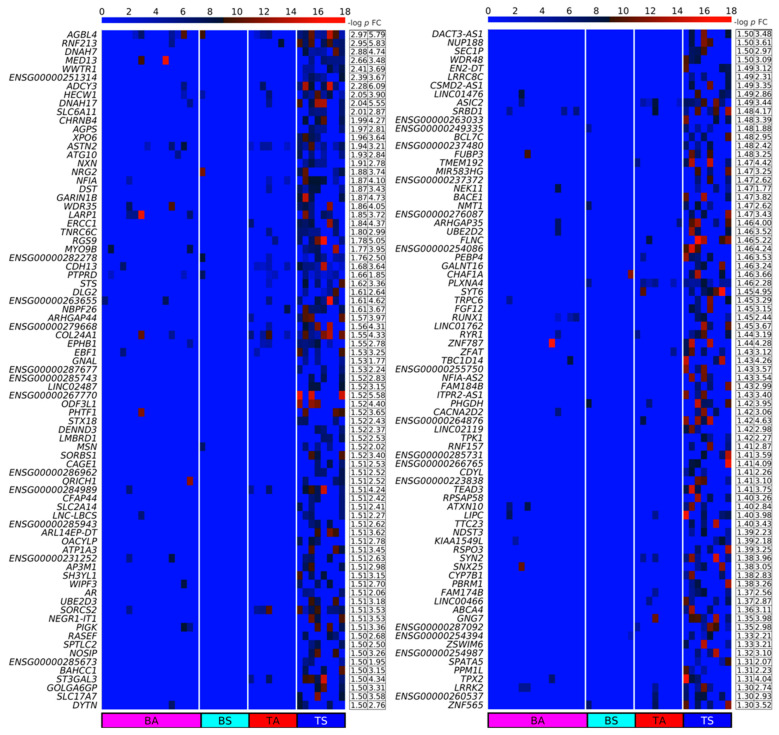
Heatmaps of the Differentially Up-Produced per Gene DNA Circles (up-DPpGCs) in the SkM of the sedentary lifestyle (TS) compared to the physically active (TA) group in decreasing order of significance. The color bar codifies the split read count of the eccDNA per gene in a log_2_ scale. Higher count corresponds to a redder color. The –log_10_ (*p*-value) and the absolute value of the log_2_ of the fold change (FC) of the DPpGCs are presented in a table to the right of the heatmap. The PpGCs in the blood samples (leukocytes) from the physically active (BA) and sedentary (BS) group are presented in the heatmap for comparison.

**Figure 5:**
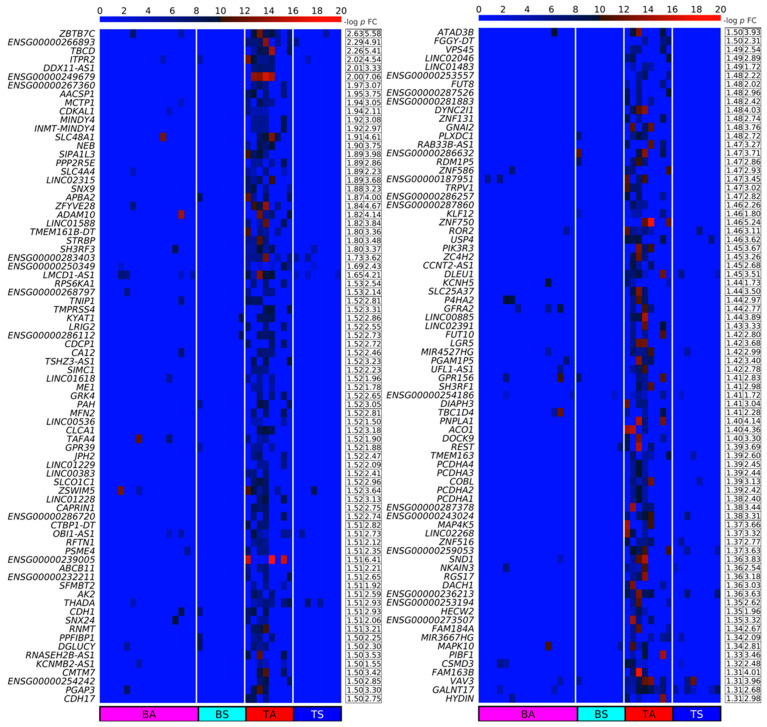
Heatmaps of the up-DPpGCs in the SkM of the physically active (TA) compared to the sedentary lifestyle (TS) group in decreasing order of significance. The color bar codifies the split read count of the eccDNA per gene in a log_2_ scale. Higher count corresponds to a redder color. The –log_10_ (*p*-value) and the absolute value of the log_2_ of the fold change (FC) of the DPpGCs are presented in a table to the right of the heatmap. The PpGCs in the blood samples (leukocytes) from the physically active (BA) and sedentary (BS) group are presented in the heatmap for comparison.

**Figure 6:**
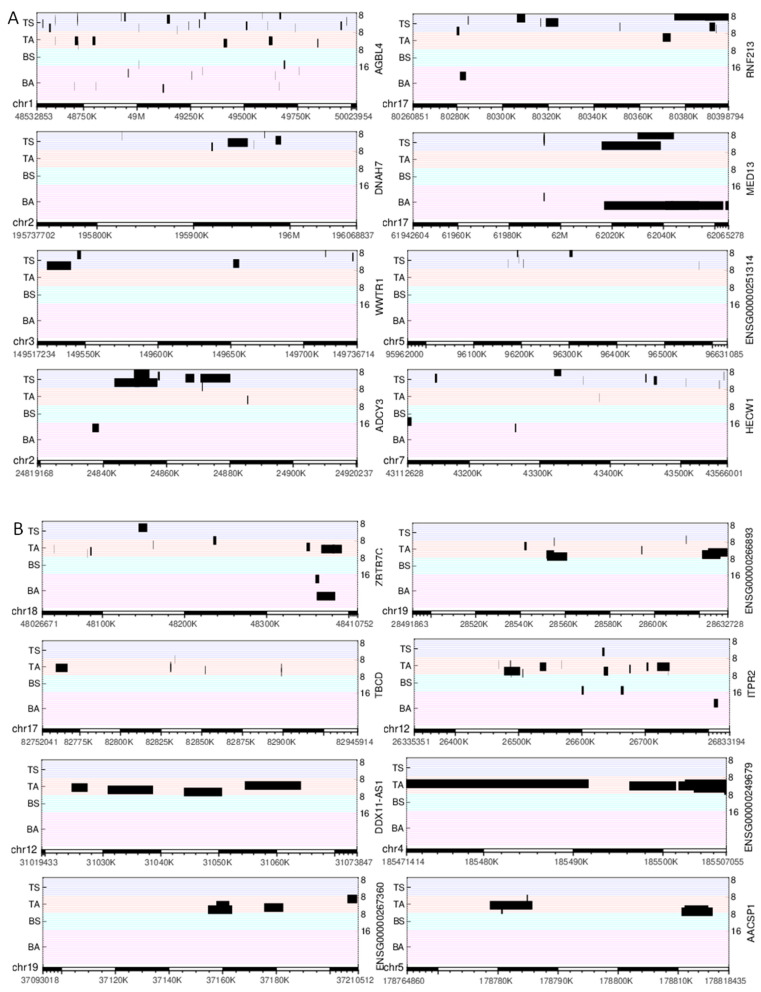
Track plots of the *loci* of the 8 top-ranked up-DPpGCs. (**A**) TS and (**B**) TA. Each horizontal line represents the length of a gene; each red line corresponds to an active (A) sample, each blue line to a sedentary (S) sample. The black bars represent the *loci* of the eccDNA.

**Figure 7:**
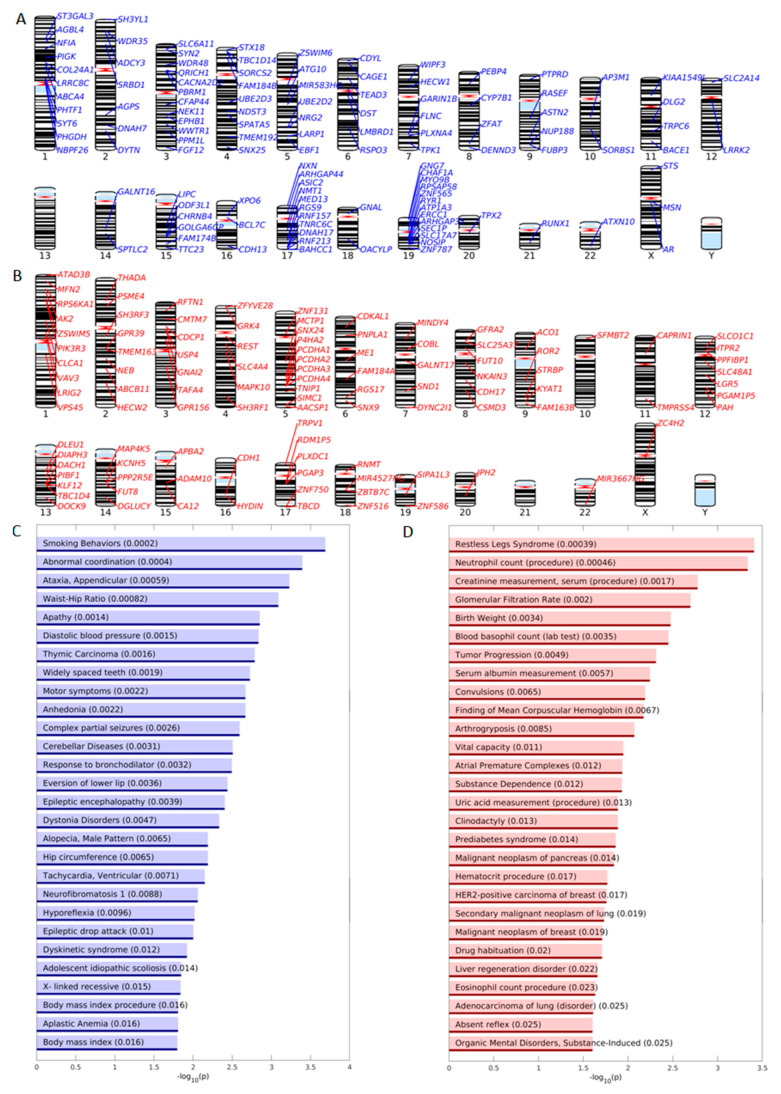
Enrichment analysis of DPpGCs. Chromosomal landscaping of the functional genomic *loci* giving rise to statistically significant up-DPpGCs in (**A**) TS and (**B**) TA. Bar plots of the –log_10_(*p*-value) of the significantly enriched DisGeNET up-DPpGCs in (**C**) TS and (**D**) TA.

We designed a method for quantifying DPpGCs based on the most important signal of the circular DNA, the number of split reads defining its circular junction. Our method assigns to each gene the number of all split reads of all eccDNA carrying fragments of this gene and normalizes for the length of the gene. We calculated the DPpGCs from the SkM of two male groups of similar age with sedentary (TS) and active (TA) lifestyle and found that a clear pattern of eccDNA origin is observed rather than underlying random events. The pairwise scatter plot of TS versus TA (Figure 3A) shows the existence of a difference in genic eccDNA between the two groups, and the volcano plot (Figure 3B) shows that there are statistically significant (significance level α = 0.05) DPpGCs. We identified 158 statistically significant up-DPpGCs in the sedentary group TS compared to the active group TA (Figure 4), and 156 up-DPpGCs in TA compared to TS (Figure 5). The *loci* of the top-ranked produced per gene eccDNA are shown in the track plots in Figure 6A,B for the top up-DPpGCs in TS and TA, and of all of them in Figures S2 and S3. The eccDNA comprising the up-DPpGCs carry fragments of the corresponding genes and these fragment sequences are rarely the same in the different samples from the same group, i.e., they are excised from different parts of the gene (Figure 6, Figures S2 and S3). The chromosomal landscape of the gene *loci* producing up-DPpGCs are shown in Figure 7A,B and differs between TS and TA. The chromosomes most enriched in DPpGCs are 17 and 19 in TS, and 5 in TA.

The top statistically significant sedentary up-DPpGC is *AGBL4* (*p*-value 0.001107), followed by *RNF213*, *DNAH7*, *MED13*, and *WWTR1*. The top active up-DPpGCs are produced by the genes *ZBTB7C* (*p*-value = 0.00236), followed by *ENSG00000266893*, *TBCD*, *ITPR2*, the long non-coding RNA *DDX11-AS1*, and *ENSG00000249679*. The functions in SkM of the top up-DPpGCs related genes and other genes of interest obtained through a literature search are shown in Tables S1 and S2 for TS and TA, respectively. Generally, the TS group genes are associated to addiction, lipid metabolism and obesity, muscle function, hypertension, and DNA damage repair, among others. The TA genes are linked with lipid metabolism, muscle growth, senescence escape, inflammation reduction, activation of the satellite cell pool, i.e., the SkM stem cells, among others.

We looked for common genes between the up-DPpGCs in TS and TA, and the significant differentially expressed genes (DEGs) induced by exercise in SkM of old mice [19], and found four genes, *ATP1A3*, *SLC17A7*, *SYN2*, and *COL24A1* from the up-DPpGCs in TS, that are related to neurotransmission and obesity (Table S1). It has been shown that voluntary exercise increases axonal regeneration from sensory neurons [23], with increase in the expression of another marker protein of synaptic vesicles, *SYN1*.

### 2.4. GSEA and DisGeNET Enrichment Analysis of the DPpGCs

We performed disease enrichment analysis using the Disease Gene Network (DisGeNET) sets and gene ontology (GO) enrichment analysis using the Gene Set Enrichment Analysis (GSEA) sets for systematic functional association of the genes related to the up-DPpGCs. *DisGeNET* is a discovery platform containing one of the largest publicly available collections of genes and variants associated to human diseases [24]. The top terms of the DisGeNET enrichment analysis for TS include the terms “Smoking Behaviors”, “Abnormal coordination”, “Ataxia, Appendicular”, “Waist-Hip Ratio”, “Apathy”, “Diastolic blood pressure” (Figure 7C). The top up-DPpGCs related gene, *AGBL4*, is associated by DisGeNET with the term “Waste-Hip Ratio” and “Body mass index” (Table S3). Genetic predisposition to higher waist-to-hip ratio adjusted for body mass index was associated with increased risk of type 2 diabetes (T2D) and coronary heart disease [25]. *ADCY3* that is among the top ten up-DPpGCs in TS is associated by DisGeNET to “Hip circumference” and “Body mass index procedure”. DisGeNET links other genes up-shedding eccDNA in TS with “Apathy” and “Anhedonia”, related to reduced motivation. Interestingly, the kynurenine aminotransferase *KYAT1* (*KAT1*) is among the top genes in TA. Exercise training induces changes in SkM that can purge the blood of kynurenine, formed during stress, and is harmful to the brain, causing depression, with the SkM’s function reminiscent of that of the kidney or the liver. The KAT enzymes in the well-trained muscles quickly convert it to kynurenic acid, resulting in a protective mechanism [26]. For TA, the top terms are “Restless Leg Syndrome”, “Neutrophil count(procedure)”, “Creatinine measurement, serum (procedure)”, “Glomerular Filtration Rate”, “Birth Weight”, “Vital capacity” (Figure 7D). Interestingly, while some studies found heritability, prenatal and birth weight as predictors of physical activity and sedentary behavior, others suggest no evidence for an association between gestational age and birth weight with sedentary behavior [27,28]. The exhaustive lists of genes associated with the enriched DisGeNET terms are given in Tables S3 and S4 for TS and TA, respectively.

The functional GSEA [29] of the sedentary SkM up-DPpGCs (Figure S4A and Table S5) include terms like “protein autoubiquitination”, “microtubule based movement”, “intrinsic hand muscle atrophy”, etc. The top TS gene *AGBL4* is associated to “retrogade axonal transport”, “neuron projection cytoplasm”, “axon cytoplasm”, ”cytoplasm region”, ”anterograde axonal transport”. The functional GSEA of the active SkM up-DPpGCs (Figure S4B and Table S6) identify as top terms “regulation of growth rate”, “negative regulation of calcium ion dependent exocytosis”, “fucose catabolic process”, “membrane protein ectodomain proteolysis”, “metal ion transport”. In muscle and other mechanically active tissue, cell membranes are constantly injured, and their repair depends on the injury induced increase in cytosolic calcium, with repair involving exocytosis of lysosomes which break down worn-out cell parts [30]. The exhaustive list of genes associated with the enriched GSEA terms are given in Tables S5 and S6 for TS and TA, respectively. The DisGeNET and GSEA enrichment analysis complementarily shed light on the relationship of the up-DPpGCs related genes with sedentary behavior or exercise and SkM, indicating that the DPpGCs identified by our method could have a functional biological readout.

### 2.5. Scaling for Gene Length Identifies Double the Number of DPpGCs

**Figure 8:**
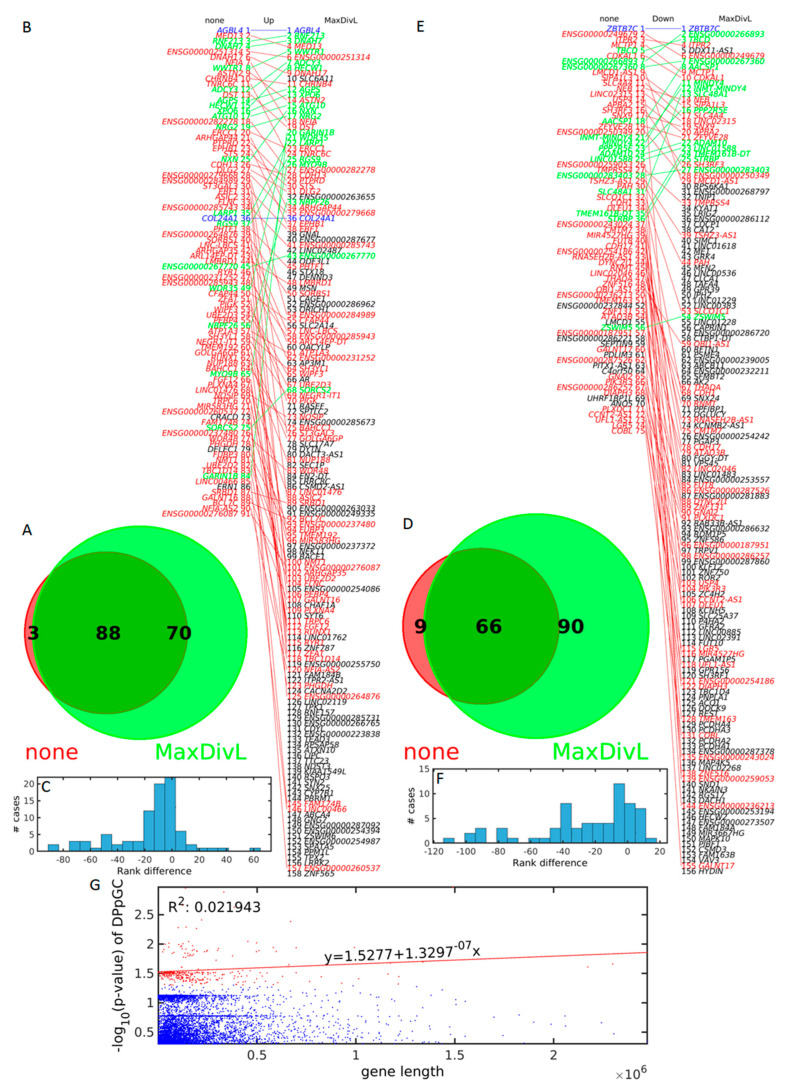
Analysis of the DPpGCs identified without (none) and with scaling for gene length (MaxDivL) methods. Venn diagram of the intersection of the up-DPpGCs identified without (red) and with (green) scaling for gene length for (**A**) TS and (**D**) TA. Comparison of the ranked up-DPpGCs without (left) and with (right) scaling for gene length (**B**) TS and (**E**) TA. The blue, red and green lines connect genes with equal, descending, and ascending ranks, respectively. The black gene names correspond to genes non-common between the two scaling methods. Histogram of the rank differences between the two scaling methods (**C**) TS (**F**) TA. (**G**) Relation between the –log_10_(*p*-value) of all the up-DPpGCs in TS and TA and the length of the underlying gene. Blue dots indicate PpGCs, red dots mark DPpGCs. The red line is the regression line of the –log_10_(*p*-value) of the statistically significant up-DPpGCs in function of the respective gene lengths.

We studied how scaling for gene length affects the number of DPpGCs by comparing the DPpGCs identified without and with scaling for gene length (Figure 8A–D). Without scaling, we obtained 91 and 75 genes in TS and TA, respectively. Applying scaling for gene length, we identified 158 and 156 in TS and TA, respectively, with the increase stronger in TA. We found that only 3 out of 91, and 9 out of 75 genes, for TS and TA, respectively, shedding DPpGCs identified with our differential analysis without scaling by gene length, were not common with the DPpGCs identified with our differential analysis with scaling by gene length. The differential analysis with scaling by gene length identifies approximately twice the number of genes identified without scaling. Interestingly, the top genes for TS and TA, *AGBL4* and *ZBTB7C*, keep their top rank without and with scaling for gene length. Additionally, all the genes with scaling until rank 30, except for *SLC6A11* with rank 10 in TS (Figure 8B), and *BPS6KA1* with rank 30 in TA (Figure 8E), are among the genes identified without scaling for gene length. Thus, the identification of the top 30 up-DPpGCs is not influenced much by the scaling. The change in rank of all the DPpGCs after scaling is shown in detail in Figure 8B,E, and the number of cases going up and down in the ranking, in Figure 8C,F.

To show that the DPpGCs scaled for gene length are not correlated to the length of the corresponding genes, we performed correlation analysis between the ranks of the genes after scaling by gene length, with the length of these genes (Figure 8G). A correlation coefficient *R*^2^ = 0.02 and a very small slope *m* = 1.130^−7^ confirms lack of such correlation.

### 2.6. RYR1 Is Transcriptomically Upregulated and up-DPpGCs Gene in Sedentary SkM

We performed differential expression of genes (DEG) analysis on the RNA-seq data from the same group of samples TA and TS, and found no significant transcriptomics difference (Figure 3C,D and Figure S5). We found 9 slightly upregulated DEGs in TA, namely *CCKB*, *LDHB*, *SNORA66*, *SNORA71C*, *PSIP1*, *SCARNA11*, *SNORA21*, *SNORA19*, *SUMO2*, and 5 upregulated DEGs in TS, namely *SLTM*, *RYR1*, *SNORD67*, *ZCCHC11*, *ANAPC11*.

Interestingly, we found that *RYR1* is both upregulated and up-DPpGCs gene in sedentary SkM. The skeletal muscle Ca^2+^ release channel, also known as ryanodine receptor type 1, is the largest ion channel protein known and is crucial for effective SkM contractile activation. Dominant and recessive mutations in *RYR1*, as well as acquired channel alterations, are the underlying cause of various SkM diseases [31]. For the gene *loci* of the eccDNA carrying *RYR1* fragments see Figure S2.

None of the transcriptomically upregulated genes are among the CPpGCs in TA or TS. They do not overlap with the list of 46 genes affecting the SkM based on meta-analysis of over 60 transcriptomics studies associated with the SkM response to exercise and inactivity in healthy individuals [32]. Additionally, we checked specifically for the expression of the genes shedding the up-DPpGCs in TS and TA and found no difference (Figure S6).

In summary, DEG analysis of the RNA-seq data found almost no difference between sedentary and active SkM. The comparison between the RNA-seq and eccDNA differential analyses results emphasizes the high sensitivity of the eccDNA in detecting the molecular fingerprints of sedentary lifestyle and physical exercise, and the ability of our DifCir method to detect differences in states with weak or no detectable transcriptomics signal.

### 2.7. Common PpGCs in the Aged Active and Sedentary SkM Add to Their eccDNA Profiles

**Figure 9:**
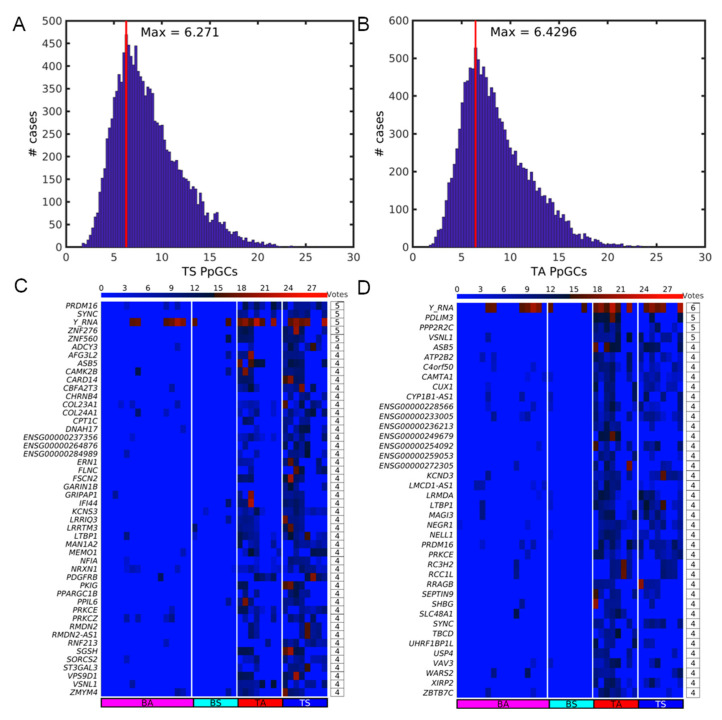
Democratic method results on the detection of common PpGCs (CPpGCs) in sedentary (TS) and active (TA) SkM. Distribution of the PpGCs for (**A**) TS and (**B**) TA. The vertical red line shows the position of the maximum of the distribution. Heatmaps of the CPpGCs in (**C**) TS and (**D**) TA. The color bars codify the split read counts of the eccDNAs per gene in a log_2_ scale. Higher count corresponds to a redder color. The number of votes of the PpGCs is presented in tables to the right of the respective heatmaps.

We applied a democratic method to look for common PpGCs (CPpGCs) in the sedentary and active SkM groups, TS and TA. To find the threshold of minimal value of PpGCs to be considered as a positive vote, we calculated the empirical distributions of the PpGCs for TS and TA (Figure 9A,B). We chose as a threshold the floor of the maxima of the distributions, in this case both equal to 6, and genes that have at least 4 votes. We found 48 CPpGCs in TS (Figure 9C) and 40 CPpGCs in TA (Figure 9D). Out of them, six, namely *ASB5*, *LTBP1*, *PKCE*, *PRDM16*, *SYNC*, and *Y_RNA*, are common CPpGCs for TS and TA, thus they are not specific for TS or TA. Rather, they seem to have general functions related to SkM (Table S7). Eleven of the CPpGCs in TS identified with the democratic method are among the DPpGCs identified with DifCir, namely *ADCY3*, *CHRNB4*, *COL24A1*, *DNAH17*, *FLNC*, *NFIA*, *RNF213*, *SORCS2*, *ST3GAL3*, *ENSG00000264876*, and *ENSG00000284989*. Eight of the CPpGCs in TA identified with the democratic method are among the DPpGCs identified with DifCir, namely *LMCD1-AS1*, *SLC48A1*, *TBCD*, *USP4*, *VAV3*, *ZBTB7C*, *ENSG00000236213*, and *ENSG00000249679*. Thus, the democratic method for identifying CPpGCs found additionally 31 (48–6–11) genes in TS and 26 (40–6–8) genes in TA producing more eccDNA than the opposite group, demonstrating that the democratic method is complementary to DifCir in the search for eccDNA fingerprint.

We looked for common genes between the CPpGCs in TS and TA, and the significant DEGs induced by exercise in SkM of old mice [19]. We found two genes *PPP2R2C* and *VSNL1* from the CPpGCs in TA, and three, *COL24A1*, *NRXN1*, and *PRKCZ* in TS (Table S7). The increased insulin-resistant SkM COL24A1 has been found to be also an up-DPpGCs in TS.

Finding CPpGCs allowed us to identify genes that produced high copy number of eccDNA in a certain number of samples of the group, TS or TA, but could not pass the requirements for statistical significance. Additionally, the calculation of CPpGCs can characterize the genic eccDNA profile of a group when another group for comparison is unavailable.

### 2.8. EccDNA in SkM Carry Whole Genes

**Figure 10:**
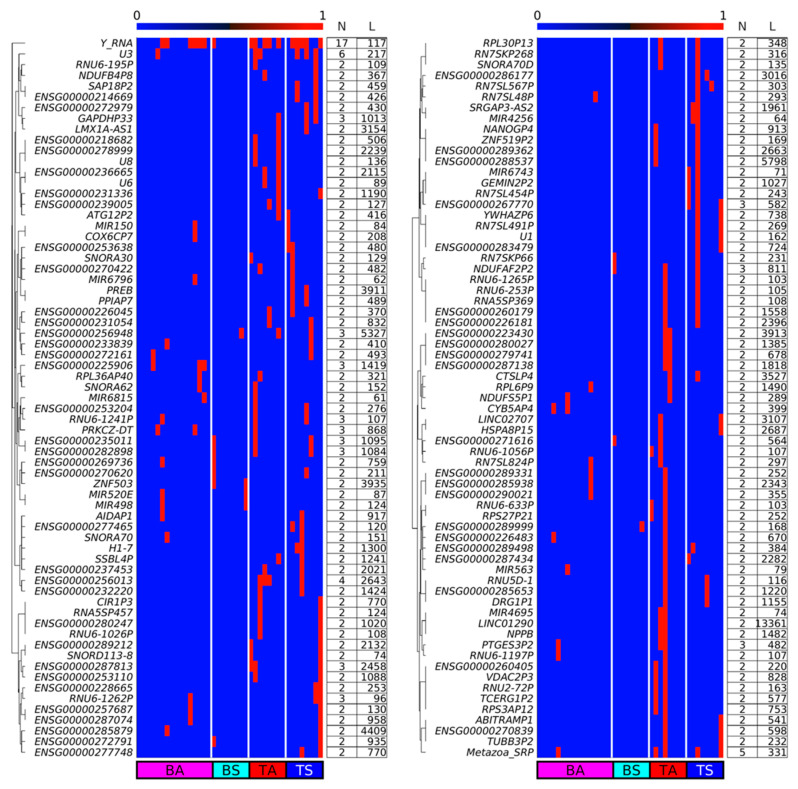
Boolean heatmap of whole genes embedded in eccDNAs in at least two samples. Numerical ‘0’ and ‘1’ correspond to the presence and absence of full genes, respectively. N: number of samples with eccDNA carrying the whole gene; L: length of the gene in bases.

We checked whether eccDNA in SkM carries full genes or only gene fragments. We found that eccDNA does carry full genes (Figure 10) of maximum length up to 13,361 bp in the case of long intergenic non-protein coding RNA 1290, *LINC01290*. Circular DNA with the same complete genes are found mostly in 2 to 3 samples. The majority of whole genes on eccDNA are pseudogenes. Among the eccDNA carrying whole genes exclusively in active SkM is the natriuretic peptide B, *NPPB* (also known as *BNP*). Natriuretic peptides enhance through exercise the oxidative capacity of human skeletal muscle, which may trigger favorable metabolic adaptations to increase fat oxidation [33], in agreement with the observation that transgenic mice overexpressing *Nppb* display higher whole-body energy expenditure and fat oxidation, lower fat mass, and higher expression of mitochondrial oxidative genes in their SkM [34]. Among the eccDNA carrying whole genes in sedentary SkM is prolactin regulatory element-binding PREB known to regulate hepatic glucose homeostasis [35].

Additionally, we checked whether some of the up-DPpGCs in TS or TA SkM carry full genes or only gene fragments. This analysis confirmed the observation that the genic eccDNA up-produced in TA and TS carry predominantly gene fragments with the exception of ENSG00000267770 in TS (Figure 10 and Figure S2) and ENSG00000239005 in TA (Figure 10 and Figure S3).

## 3. Discussion

In a milestone paper, Wang et al. [12] observed that eccDNAs map across the entire genome in a close to random manner suggesting a biogenesis mechanism of random ligation of genomic DNA fragments. Such a pattern of eccDNA mapping in a random manner across the genome is indeed observed in the aged SkM eccDNA purified data [3]. Furthermore, the groups of sedentary and active aged SkM are not statistically significant different neither in the numbers nor in the frequency of the length distributions of their unique eccDNA sequences circularized from both genic and intergenic *loci* of the genome. Interestingly, thanks to our DifCir methods we found a pattern of difference between the genic profiles of the eccDNA of groups of sedentary and active aged SkM, i.e., we found that the distribution of the eccDNA circularized from gene regions is not random. The differential eccDNA in the sedentary SkM group are circularized from genes associated to addiction, lipid metabolism and obesity, muscle function, hypertension, and DNA damage repair, while the active lifestyle characteristic eccDNA are circularized from genes linked with lipid metabolism, muscle growth, senescence escape, inflammation reduction, and activation of the satellite cell pool. Wang et al. [12] demonstrated that eccDNA are apoptotic products with high innate immunostimulatory activity. Though it has been shown in the literature and our own RNA-seq analysis confirmed that the difference at transcriptomics level between sedentary and exercised older SkM is small, we found a gene, *RYR1*, which excised differentially eccDNA in the sedentary group was also among the few upregulated genes in the sedentary SkM. The identified up-DPpGCs in sedentary and active SkM seemingly do not follow a pattern and predominantly do not encode full length genes. In contrast to the long cancer ecDNAs carrying whole genes and their multiple copies, here we identify differences in production of eccDNA carrying mostly gene fragments. Therefore, it seems unlikely that this is a coordinated response to increase transcription. Conversely, it seems to be rather a compensatory mechanism to repair and ameliorate an abnormal condition. Therefore, the positive correlation between increased eccDNA production from a gene and increased transcription might be wrongly interpreted as a cause and a result while the direction of the relationship might be opposite.

The interpretation of the short-read sequenced purified eccDNA data from sedentary and active SkM shows that differential eccDNA analysis based on the split-read signal of the eccDNA can function as a biomarker. We found for the first-time differences between the eccDNA of two groups, while none were detected in the first analysis of the data [3] where the purified eccDNA Circle-Seq and RNA-Seq data from the two groups was generated. The differential genic eccDNA comparison between aged SkM of a sedentary and active group is interesting and needed since the numbers of unique eccDNAs and the length distributions of the eccDNA species are very similar, while number and length characteristics cannot serve as a biomarker as for example where they are used for nuclease activity and systemic lupus erythematosus [36].

Currently eccDNA differential analysis relies on initial selection of unique circles mapped with high confidence. Our method differs to others such as in [37] in that they count the number of unique eccDNA mapped to a gene, while we count the number of split-reads mapped to a gene, an approach similar to the count units in DEG analysis in RNA-seq. We assume that the chromosomal DNA of many single cells in a sample might shed the same unique sequence eccDNA and we use the split-read count as a number of eccDNA. Conversely, others count the unique eccDNA types in a sample, and use the split-read number to empirically decide whether a sequence is a confident unique eccDNA species.

Another approach of differential analysis of short-sequence eccDNA enriched data is to find the differential “expression” of the eccDNA between two conditions through employing established methods for differential analysis of RNA-seq data as edgeR [38] or cuffdiff [39], and it has been used in a growing number of works [18,40,41]. Anyway, these works identify genes with differential “expression” rather than differentially “expressed” eccDNA since the concordant read coverage cannot be directly related to the copy number of the amplified eccDNA. Generally, a gene that has higher “expression” owes that expression to many unique eccDNA sequences with different lengths, and with the longer eccDNA contributing more to the cumulative coverage of the gene. Moreover, it is very rare that the same eccDNA sequence, i.e., exactly the same eccDNA specie, can be found in all the biological replicates of a group. In contrast, our differential method does not favor genes that shed longer eccDNA since it counts the signal of the circular junction of the eccDNA, the split read. It is based on identifying genes with higher “copy number” of eccDNA in a group compared to its control. Importantly, DifCir does not need to merge or select high quality eccDNA, with involved parameters affecting the number of unique eccDNA. It can directly use the split reads from sequenced eccDNA purified data as a measure of eccDNA signal.

DifCir suffers from the general limitations of the purified eccDNA Circle-Seq data. Major issues are differences between samples that arise due to library preparation and sampling. EccDNA extraction is a protocol with many steps which can contribute to the variation in eccDNA detection between samples, the two most obvious being differential PCR amplification of fragments in different libraries and sequencing depth. The second source of variation comes from the fact that different samples will be sequenced to different depths. Both of these factors influence which eccDNA fragments are detected, and consequently, which are being quantified. Additionally, it is dependent on the gene annotation.

In summary, our computational method DifCir succeeded in finding a differential molecular fingerprint in the SkM of lifelong sedentary and active people when the other molecular method RNA-seq and the eccDNA computational analysis in [3] failed.

## 4. Methods

### 4.1. Characteristics of Participants and eccDNA Purification

We used purified eccDNA short-read sequenced data from skeletal muscle (SkM) and blood from 16 male participants, healthy but physically inactive (*n* = 8, age 62.8 ± 1.3 years) with lifelong sedentary lifestyle who exercised at most once per week, and physically active (*n* = 8, age 62.1 ± 1.4 years) who exercised more than three times per week or more throughout their life. Selection of men in the two groups was based on questionnaires. The reported physical activity differed over the years for several of the participants and included soccer, bicycling, hiking, running, gymnastics, handball, badminton, military training, fitness, swimming, tennis, boxing, track and field, and ice hockey. Moreover, the lifelong physically active subjects trained for at least 5 h/week at the time of the experiment. While height and weight were similar between the two groups, body fat percentage was 39% higher with a corresponding 13% lower percentage of lean mass in the inactive than the active men. Cycling endurance was 41% lower in the inactive group than the active indicating a large difference in overall physical fitness. Three physically inactive subjects and one physically active subject were smokers, while all other subjects were nonsmokers [21]. All odd numbers are samples from physically inactive men; all even numbers from active men. For comparison, we used data from blood samples (leukocytes) from the same participants [3], denoted with B in the heatmaps. On the day of the experiment, subjects arrived at the laboratory late afternoon (5 PM) having fasted at least 2 h prior to experiment. Muscle biopsies were collected from the *vastus lateralis*, transferred into liquid nitrogen, and stored at –80 °C; tissues were fractionated at −20 °C and aliquots of 50–100 mg were sliced into thin pieces with a sterile scalpel and air-dried at room temperature for 1 h before weighing 6 mg of dry tissue per sample [3]. The Circle-Seq protocol of Møller [11] was used for isolation and physical enrichment of the chromosome-derived eccDNA, consisting of concise alkaline treatment and gentle gravity flow through an ion-exchange column, by which eccDNAs were enriched in the eluate fraction; eccDNAs were enzymatically isolated by extensive Plasmid-Safe DNase digestion of linear chromosomes and further enriched by φ29 rolling circle amplification. The purified fragmented DNA was paired-end (2 × 100 bp) sequenced. In this study, we used the Circle-Seq data presented in [3].

### 4.2. EccDNA Mapping and Quantification of the Produced per Gene eccDNA (PpGCs)

We mapped the eccDNA using Circle_finder [2,5] using as arguments the hg38 built of the genome and minNonOverlap between two split reads equal to 10. We filtered out mitochondrial eccDNA and eccDNA with lengths greater than *L_max_*_,_ using *L_max_* = 100 Kb. Previous analysis found that the majority of eccDNA was smaller than 25Kb [3]. Anyway, we assessed the eccDNA sizes found with Circle_finder, with the histograms of all samples presented in Figure S7 and found that 100 Kb is a cutoff for all short circles. Next, we merged clusters of circles within a distance smaller than *D_min_* = 10 b and added up the split reads of the merged circles. We filtered out the circles with less than *JT_min_* = 2 split reads. We annotated the circles using bedtools intersect [42] on the resulted eccDNA bed files with a GencodeHuman38 bed file with the gene coordinates. Then we unified the circles based on genes, i.e., all the eccDNA that carry the same gene or fragment of this gene were grouped together. We scaled by gene length, multiplying each Produced per Gene eccDNA *i* (PpGC*_i_*) by a scale factor *L_Max_*/*L_i_*, where *L_Max_* is the length of the longest gene found in the dataset, and *L_i_* is the length of the gene *i*. Finally, we performed data equalization by the log_2_(PpGC + 1) transform of the quantified PpGCs.

### 4.3. Differential Analysis for Identifying Differentially Produced per Gene DNA Circles (DPpGCs)

We calculated the average values for each group of replicates. We selected the DPpGCs whose absolute value of difference of mean values between the two groups was less than a selection threshold θ_DPpGC_ = 1 of fold change (FC) in log_2_ scale. We selected the statistically significant DPpGCs using the Student’s *t*-test with a significance threshold α_DPpGC_ = 0.05.

### 4.4. Democratic Method for Finding Common PpGCs in a Group

Similar to that previously reported in transcriptomics [43] and DNA methylomics [44], we implemented a democratic method for circulomics data. To find the threshold of a minimal value of PpGCs to be considered as a positive vote, we calculated the empirical distribution of the group. We chose as a threshold, Max, the floor of the maximum of the distribution of the PpGCs, i.e., Max = floor(f(PpGC)), where f(PpGC) is the distribution of PpGC. Finally, we selected the genes that had at least 4 votes in the group.

### 4.5. RNA-Seq Data Analysis

We use the RNA-Seq data presented in [3]. We mapped the RNA-seq data to the human reference genome GRCh38 using HISAT [45]. Cufflinks was used to assemble the mapped reads into possible transcripts and to generate a transcriptome assembly [46]. We merged the expression results into a single text file and used it in the downstream analysis in Matlab. We equalized the data and stabilized them through the log_2_ transform of the data plus one; calculated the average values for each group of replicates; selected the DEGs whose absolute value of difference of mean values between the two groups was less than a selection threshold θ_DEG_ = 1 of FC in log_2_ scale; selected the statistically significant DEGs using the Student’s *t*-test with a significance threshold α_DEG_ = 0.05.

## 5. Conclusions

We designed a method and pipeline DifCir to find the eccDNA difference between two groups of samples based on the split-read signal and quantifying the Differentially Produced per Gene DNA Circles (DPpGCs). The proposed DPpGCs identification method is crucial in the use short-read sequenced purified eccDNA data in precision medicine for monitoring and prediction of cancer development, inflammatory disease state, and as a diagnostic marker in different skeletal muscle disorders. It allows fast identification of genic regions shedding more eccDNA in the given state that could be potential early markers of that state. Using DifCir, we succeeded in identifying differential genic eccDNA profiles of two groups of aged individuals of the same sex with different exercise life choices whose otherwise general eccDNA characteristics as number and length distribution, are very similar, and show only very subtle differences at transcriptomics level. Last but not least, the results support the importance of lifestyle choices on the gene level.

## Data Availability

Sequence data from Circle-Seq and RNA-Seq experiments from skeletal muscle was downloaded from the Sequence Read Archive. BioSample accession IDs for DNA Circle-Seq: SAMN08054900 to AMN08054941. Bioproject ID for RNA-seq PRJNA392413. The computational method is implemented in Matlab into a software tool DifCir available at https://github.com/MarcosArauzoBravo/DifCir/.

## Supplementary Materials

The following supporting information can be downloaded at: https://www.mdpi.com/article/10.3390/ijms24032736/s1 [47,48,49,50,51,52,53,54,55,56,57,58,59,60,61,62,63,64,65,66,67,68,69,70,71,72,73,74,75,76,77,78,79,80,81,82,83,84,85,86,87,88,89,90,91,92,93].

## Abbreviations

Circle-Seq	Circular DNA purification protocol
CPpGCs	Common Produced per Gene DNA Circles
DEG	Differentially Expressed Genes
DifCir	Method and tool for Differential analysis of Circular DNA
DisGeNET	Disease Gene Network
DPpGCs	Differentially Produced per Gene DNA Circles
ecDNA	Long (>1 Mb) oncogenic extrachromosomal circular DNA
eccDNA	Extrachromosomal circular DNA
FC	Fold Change
GO	Gene Ontology
GSEA	Gene Set Enrichment Analysis
MtDNA	Mitochondrial DNA
PpGCs	Produced per Gene DNA Circles
sem	Standard Error of the Mean
SkM	Skeletal Muscle
T2D	Type 2 Diabetes
WGS	Whole Genome Sequencing
