# Supplementary material for: Skeletal Muscles of Sedentary and Physically Active Aged People Have Distinctive Genic Extrachromosomal Circular DNA Profiles
Source: Int J Mol Sci. 2023 Feb 1;24(3):2736. doi: 10.3390/ijms24032736 (PMC9917053; doi:10.3390/ijms24032736)
Supplement: Supplementary file 1 [file ijms-24-02736-s001.zip › ijms-2101316-supplementary.pdf]

Skeletal muscles of sedentary and physically active aged people have distinctive genic extrachromosomal circular DNA profiles

Supplementary Material

Daniela Gerovska[1*] and Marcos J. Araúzo-Bravo[1,2,3,4,5*]

[1] Computational Biology and Systems Biomedicine, Biodonostia Health Research Institute, Calle Doctor Begiristain s/n, 20014, San Sebastian, Spain

[2] Basque Foundation for Science, IKERBASQUE, Calle María Díaz Harokoa 3, 48013, Bilbao, Spain

[3] CIBER of Frailty and Healthy Aging (CIBERfes), Madrid, Spain

[4] Max Planck Institute for Molecular Biomedicine, Computational Biology and Bioinformatics, Röntgenstr. 20, 48149, Münster, Germany

[5] Department of Cell Biology and Histology, Faculty of Medicine and Nursing, University of Basque Country (UPV/EHU), 48940 Leioa, Spain

* Corresponding authors: Daniela Gerovska: daniela.gerovska@biodonostia.org, Marcos J. Araúzo-Bravo: mararabra@yahoo.co.uk

Daniela Gerovska ORCID: orcid.org/0000-0003-0671-4277

Marcos J. Araúzo-Bravo ORCID: orcid.org/0000-0002-3264-464X

Supplementary Tables

Table S1. Up-DPpGC-associated genes for the TS group and their function in SkM.

**Gene**	**Function**
AGBL4 (CCP4)<br>AGBL carboxypeptidase 4	Obesity/BMI-associated locus with expression enrichment in the brain regions insula and substantia nigra, involved in addiction and reward (Ndiaye *et al.*, 2020); member of CCP enzyme family modifying MLCK (Rogowski *et al.*, 2010), with the smooth muscle smMLCK isoform specifically required to regulate smooth muscle contractility (Chen *et al.*, 2013).
RNF213 (mysterin)<br>ring finger protein 213	Role in insulin-regulating pathways (Sarkar *et al.*, 2021); necessary for SkM myogenesis (Kotani *et al.*, 2015).
DNAH7<br>dynein axonemal heavy chain 7	Adipogenesis-associated gene (Söhle *et al.*, 2012).
MED13<br>mediator complex subunit 13	Role in governance of glucose metabolism and control of hepatic lipid accumulation by SkM (Amoasii *et al.*, 2016).
WWTR1 (TAZ)<br>WW domain containing transcription regulator 1	Downstream effector in the Hippo pathway, plays key role in myoblast proliferation and enhances myogenic differentiation (Sun et al., 2017); Myoblast proliferation and differentiation is altered in aging, associated with low physical activity, which may alter YAP/TAZ levels in myoblasts, cause stiff extracellular matrix and induce further fibrosis through YAP/TAZ (Setiawan *et al.*, 2021).
CTD-2337A12.1<br>uncharacterized LOC101929710	rs6232 associated with a higher risk of obesity and insulin resistance (https://selfdecode.com/app/snp/rs6232/).
ADCY3<br>adenylate cyclase 3	ADCY3 variants are risk of obesity and T2D (Grarup *et al.*, 2018).
HECW1<br>HECT, C2 and WW domain containing E3 ubiquitin protein ligase 1	Identified in muscle-invasive transitional cell carcinoma (Pan *et al.*, 2016).
DNAH17<br>dynein axonemal heavy chain 17	Involved in fatty tissue biology, KD causes reduced neutral lipid accumulation (Söhle *et al.*, 2012).
SLC6A11<br>solute carrier family 6 member 11	Genetic perturbation of SLC16A11 induces changes in fatty acid and lipid metabolism associated with increased T2D risk (Rusu *et al.*, 2017).
**...**	**Upper row genes (10) in rank order; Lower row genes in alphabetic order**
ATP1A3<br>ATPase Na+/K+ transporting subunit alpha 3	Transmembrane ion-pump maintaining the electrochemical gradients of Na and K ions across the plasma membrane, important for electrical excitability of nerve and muscle.
BACE1<br>beta-secretase 1	Sustain muscle spindles and maintain motor coordination (Cheret *et al.*, 2013).
COL24A1<br>collagen type XXIV alpha 1 chain	Increased in insulin-resistant SkM and adipose tissue (Weng *et al.*, 2020).
NEK11<br>NIMA related kinase 11	Linked with DNA replication and response to genotoxic stress, central role in the G2/M checkpoint and the DNA damage response (Peres de Oliveira *et al.*, 2020).
NMT1<br>N-myristoyltransferase 1	Overexpressed and hypermethylated in visceral adipose tissue of severely obese individuals with Metabolic Syndrome; SNPs associated with altered plasma lipid levels, increased inflammation markers and blood pressure among severely obese patients (Bégin *et al.*, 2015).
SLC17A7 (*VGLUT1*)<br>solute carrier family 17 member 7	Associated with the membranes of synaptic vesicles and functions in glutamate transport.
SYN2<br>synapsin II	Marker protein of synaptic vesicles filling the nerve terminal of neurons (Thiel, 1993).
UBE2D2<br>ubiquitin conjugating enzyme E2 D2	Role in atrophying SkM (Polge *et al.*, 2015)

Table S2. Up-DPpGC-associated genes for the TA group and their function in SkM.

**Gene**	**Function**
ZBTB7C<br>zinc finger and BTB domain containing 7C	Proto-oncoprotein, controls cell cycle and glucose, glutamate, and lipid metabolism; KO or KD of *Zbtb7c* decreases adipose tissue mass in aging mice (Choi *et al.*, 2021).
ENSG00000266893	Long intervening noncoding RNA
TBCD<br>tubulin folding cofactor D	Significantly hypermethylated in aged muscle tissue (Zykovich *et al.*, 2014)
ITPR2<br>inositol 1,4,5-trisphosphate receptor type 2	Necessary for mitochondrial calcium uptake, KD associated to senescence escape, indicating the role of mitochondrial calcium accumulation in senescence induction (Wiel *et al.*, 2014); promotes cellular senescence and aging (Ziegler *et al.*, 2021)
DDX11-AS1<br>DEAD/H-box helicase 11 Antisense RNA 1	Long non-coding RNA; DDX11 involved in DNA replication and sister chromatid cohesion, possible role in DNA repair (Bharti *et al.*, 2014).
AACSP1<br>acetoacetyl-CoA synthetase pseudogene 1	Associated with cardiorespiratory fitness and endurance performance (Zhao *et al.*, 2020)
MCTP1<br>*multiple C2 and transmembrane domain containing 1*	Promotes lipid droplet biogenesis (Joshi *et al.*, 2021); SNP associated with physical activity (Kim *et al.*, 2014)
**...**	**Upper row genes in rank order; Lower row genes in alphabetic order**
AACSP1<br>acetoacetyl-CoA synthetase pseudogene 1	rs12518860 associated with cardiorespiratory endurance (Zhao *et al.*, 2020)
ADAM10<br>ADAM metallopeptidase domain 10	Indispensable for maintenance of the muscle satellite cell pool (Mizuno *et al.*, 2019)
CDCP1 (*CD318*)<br>CUB domain containing protein 1	Expressed in satellite cells (Uezumi *et al.*, 2016); potential to preserve and recover SkM mass and function in cachexia, sarcopenia, and neuromuscular diseases.
CDKAL1<br>CDK5 regulatory subunit associated protein 1 like 1	T2D susceptibility gene; regulates mitochondrial function in adipose tissue, mRNA levels reduced in adipose tissue of obese mice (Palmer *et al.*, 2017).
KYAT1 (*KAT1*)<br>kynurenine aminotransferase 1	Catalyzes the production of kynurenic acid which activates GPR35/AMPK and SIRT6 pathways and reduces inflammation and insulin resistance in SkM (Jung *et al.*, 2020).
MFN2<br>mitofusin 2	Adult SkM deletion impedes exercise performance and training capacity (Bell *et al.*, 1985).
NEB<br>nebulin	Regulates thin filament length, contractility, and Z-disk structure *in vivo*. Myopathic changes in the nebulin KO model include depressed contractility, loss of myopalladin from the Z-disk, and dysregulation of genes involved in calcium homeostasis and glycogen metabolism (Witt *et al.*, 2006)
PPP2R5E<br>protein phosphatase 2 regulatory subunit B'epsilon	Implicated in the negative control of cell growth and division. Gene affected by exercise (Radom-Aizik *et al.*, 2009).
ROR2<br>receptor tyrosine kinase like orphan receptor 2	Roles in morphogenesis and formation of the musculoskeletal system during embryonic development and its regeneration and maintenance in adults (Kamizaki *et al.*, 2021).
RPS6KA1<br>ribosomal protein S6 kinase A1	Influences healthy mammalian life-span; its therapeutic manipulation mimics caloric restriction and protect against diseases of aging (Selman *et al.*, 2009)
STRBP<br>spermatid perinuclear RNA binding protein	Highly expressed in testis and brain; negatively correlated with age and aging (Li *et al.*, 2019).
TNIP1<br>TNFAIP3 interacting protein 1	Repressor of inflammatory signaling and factor in autoimmune diseases (Shamilov *et al.*, 2018).
TSHZ3-AS1<br>TSHZ3 antisense RNA 1	Expressed in quiescent satellite cells of adult mouse SkM; regulates SkM differentiation (Faralli *et al.*, 2011).

Table S3. Functional enrichment analysis of DPpGCs overrepresented in TS based on the DisGeNET annotation. Number of genes in the signal set 158. Number of genes in the background set 10771.

**Count**	***p*-value**	**DisGeNET**	**Genes**
11/139	$2.050e^{-4}$	Smoking Behaviors	*ASIC2, CDH13, CHRNB4, DYTN, HECW1, MYO9B, PBRM1, PTPRD, RSPO3, RUNX1, SNX25*
4/19	$4.033e^{-4}$	Abnormal coordination	*ATP1A3, ATXN10, CACNA2D2, LMBRD1*
5/35	$5.908e^{-4}$	Ataxia, Appendicular	*ATP1A3, ATXN10, CACNA2D2, CYP7B1, FGF12*
15/267	$8.168e^{-4}$	Waist-Hip Ratio	*AGBL4, ASTN2, CDH13, COL24A1, DLG2 ,DNAH17, EPHB1, MYO9B, PBRM1, RNF157, RSPO3, RUNX1, SPATA5, WDR35, ZFAT*
4/26	$1.413e^{-3}$	Apathy	*AR, ATXN10, LRRK2, MSN*
13/228	$1.462e^{-3}$	Diastolic blood pressure	*ADCY3, ASTN2, CACNA2D2, CDH13, DNAH7 ,EBF1 ,LINC01762, LIPC LRRC8C, MYO9B, NXN, PTPRD, UBE2D3*
4/27	$1.636e^{-3}$	Thymic Carcinoma	*AR, ERCC1, PBRM1, SORBS1*
4/28	$1.882e^{-3}$	Widely spaced teeth	*ATP1A3,SPATA5, WDR35, ZSWIM6*
4/29	$2.153e^{-3}$	Anhedonia	*ATP1A3, CDYL, CHRNB4, SLC17A7*
4/29	$2.153e^{-3}$	Motor symptoms	*AR, ATP1A3, LRRK2, SLC17A7*
5/48	$2.554e^{-3}$	Complex partial seizures	*ATXN10, CHRNB4, ERCC1 ,SLC17A7, SYN2*
4/32	$3.127e^{-3}$	Cerebellar Diseases	*ATP1A3, CYP7B1, FLNC, SLC17A7*
6/71	$3.206e^{-3}$	Response to bronchodilator	*CDH13, CHRNB4,DLG2, FGF12, PPM1L, PTPRD*
5/52	$3.647e^{-3}$	Eversion of lower lip	*ERCC1, PHGDH, QRICH1, WDR35, ZSWIM6*
6/74	$3.949e^{-3}$	Epileptic encephalopathy	*ATP1A3 ,CACNA2D2, FGF12 ,PHGDH, SPATA5, ST3GAL3*
5/55	$4.662e^{-3}$	Dystonia Disorders	*ATP1A3 ,DST, GNAL ,LRRK2, RGS9*
4/39	$6.490e^{-3}$	Hip circumference	*ADCY3, EBF1, RSPO3, ZFAT*
4/39	$6.490e^{-3}$	Alopecia, Male Pattern	*AR, EBF1, RUNX1 ,STS*
4/40	$7.112e^{-3}$	Tachycardia, Ventricular	*FGF12, FLNC, RYR1, SEC1P*
7/112	$8.750e^{-3}$	Neurofibromatosis 1	*AR, ASIC2 ,EPHB1 ,NFIA ,RNF213,RUNX1 ,RYR1*
7/114	$9.606e^{-3}$	Hyporeflexia	*AR, ATXN10, FGF12 ,FLNC, QRICH1, RYR1, ZSWIM6*
4/44	$9.992e^{-3}$	Epileptic drop attack	*CHRNB4, PHGDH, SLC17A7 SYN2*
7/119	$1.201e^{-2}$	Dyskinetic syndrome	*ATP1A3, ATXN10, FGF12, LRRK2, RGS9, SPATA5, ST3GAL3*
14/332	$1.428e^{-2}$	Adolescent idiopathic scoliosis	*AR, ASIC2, ASTN2, ATXN10, CDH13, COL24A1, DLG2, GALNT16, LINC02119, LIPC, PTPRD, SORCS2, STX18, SYN2*
4/49	$1.454e^{-2}$	X- linked recessive	*ABCA4, AR, MSN, STS*
4/50	$1.559e^{-2}$	Aplastic Anemia	*AR, MSN, RUNX1, SORBS1*
4/50	$1.559e^{-2}$	Body mass index procedure	*ADCY3, COL24A1, RSPO3, SYN2*
20/536	$1.597e^{-2}$	Body mass index	*ADCY3, AGBL4, ASIC2, CDH13, CDYL, CHAF1A, CHRNB4, COL24A1, DLG2, EBF1, EPHB1, GALNT16, LIPC, MIR583HG, PBRM1, PTPRD, SORBS1, SYN2, TNRC6C, ZSWIM6*

Table S4. Functional enrichment analysis of DPpGCs overrepresented in TA based on the DisGeNET annotation. Number of genes in the signal set 155. Number of genes in the background set 10771.

**Count**	***p*-value**	**DisGeNET**	**Genes**
5/41	$3.920e^{-4}$	Restless Legs Syndrome	*MFN2, PCDHA3, RNMT, SLC25A37, ZC4H2*
6/63	$4.609e^{-4}$	Neutrophil count (procedure)	*PCDHA1, PCDHA2, PCDHA3, PCDHA4, PIK3R3, THADA*
5/56	$1.674e^{-3}$	Creatinine measurement, serum (procedure)	*DACH1, JPH2, LMCD1-AS1, SIPA1L3, THADA*
9/170	$2.010e^{-3}$	Glomerular Filtration Rate	*CA12, DACH1, JPH2, NEB, PIK3R3, SIMC1, SIPA1L3, SND1, THADA*
6/92	$3.355e^{-3}$	Birth Weight	*CDKAL1, ITPR2, ME1, OBI1-AS1, SLC25A37, SND1*
7/122	$3.550e^{-3}$	Blood basophil count (lab test)	*P4HA2, PCDHA1, PCDHA2, PCDHA3, PCDHA4, THADA, TNIP1*
33/1178	$4.870e^{-3}$	Tumor Progression	*ADAM10, AK2, ATAD3B, CA12, CDCP1, CDH1, CDH17, DACH1, DIAPH3, DLEU1, FUT8, GNAI2, GPR39, KCNH5, LGR5, LINC00536, LRIG2, MAP4K5, MFN2, PIBF1, PIK3R3, PLXDC1, PPFIBP1, REST, RGS17, ROR2, RPS6KA1, SND1, SNX9, TMPRSS4, TRPV1, VAV3, ZC4H2*
9/199	$5.711e^{-3}$	Serum albumin measurement	*ABCB11, ADAM10, CDKAL1, ITPR2, NKAIN3, PCDHA1, PCDHA2, PCDHA3, PCDHA4*
4/50	$6.477e^{-3}$	Convulsions	*ME1, MFN2, REST, TBCD*
11/276	$6.730e^{-3}$	Finding of Mean Corpuscular Hemoglobin	*CDH1, CDKAL1, ITPR2, NEB, PIBF1, PIK3R3, RPS6KA1, SLC25A37, TBC1D4, THADA USP4*
4/54	$8.519e^{-3}$	Arthrogryposis	*NEB, ROR2, TBCD, ZC4H2*
9/222	$1.131e^{-2}$	Vital capacity	*CCNT2-AS1, DIAPH3, DLEU1, JPH2, LMCD1-AS1, PCDHA1, PCDHA2, PCDHA3, PCDHA4*
4/59	$1.162e^{-2}$	Atrial Premature Complexes	*CDH1, LGR5, SND1, TRPV1*
5/88	$1.175e^{-2}$	Substance Dependence	*CDCP1, CSMD3, FAM184A, KLF12, RGS17*
6/122	$1.303e^{-2}$	Uric acid measurement (procedure)	*CDKAL1, DACH1, INMT-MINDY4, LINC01229, MINDY4, SIPA1L3*
4/61	$1.304e^{-2}$	Clinodactyly	*CDH1, PAH, PGAP3, ROR2*
4/62	$1.379e^{-2}$	Prediabetes syndrome	*CDKAL1, DACH1, MFN2, TBC1D4*
22/772	$1.437e^{-2}$	Malignant neoplasm of pancreas	*ACO1, ADAM10 ,CDCP1, CDH1, CDH17, CDKAL1, CLCA1, DACH1, GFRA2, KLF12, MAP4K5, MFN2, P4HA2, PIK3R3, PPFIBP1, REST, ROR2, RPS6KA1, SLC25A37, TNIP1, TRPV1, VAV3*
4/66	$1.706e^{-2}$	Hematocrit procedure	*CCNT2-AS1, DLEU1, HYDIN, TMEM163*
5/97	$1.738e^{-2}$	HER2-positive carcinoma of breast	*ADAM10, CA12, CDCP1, PIK3R3, SFMBT2*
13/401	$1.853e^{-2}$	Secondary malignant neoplasm of lung	*ADAM10, CA12, CAPRIN1, CDCP1, CDH1, CDH17, FUT8, P4HA2, RPS6KA1, SLC25A37, SND1, TMPRSS4, VAV3*
49/2079	$1.947e^{-2}$	Malignant neoplasm of breast	*ABCB11, ADAM10, ATAD3B, CA12, CAPRIN1, CDCP1, CDH1, CDH17, CDKAL1, CLCA1, DACH1, DIAPH3, DLEU1, FUT8, GNAI2, GPR39, GRK4, LGR5, LINC01483, MAPK10, MFN2, MINDY4, P4HA2, PAH, PGAM1P5, PGAP3, PIBF1, PIK3R3, PPP2R5E, REST, RFTN1, RGS17, RNMT, ROR2, RPS6KA1, SLC25A37, SLC48A1, SND1, SNX9, STRBP, TAFA4, TMPRSS4, TNIP1, TRPV1, USP4, VAV3, ZNF131, ZNF516, ZSWIM5*
5/100	$1.959e^{-2}$	Drug habituation	*CDCP1, CSMD3, FAM184A, KLF12, SND1*
5/103	$2.199e^{-2}$	Liver regeneration disorder	*ADAM10, CDH1, LGR5, MAPK10, PAH*
9/251	$2.328e^{-2}$	Eosinophil count procedure	*DLEU1, LINC01229, LINC02391, P4HA2, PIK3R3, SIPA1L3, THADA, TNIP1, VAV3*
20/724	$2.459e^{-2}$	Adenocarcinoma of lung (disorder)	*AK2, ATAD3B, CA12, CDCP1, CDH1, DACH1, DIAPH3, LMCD1-AS1, MFN2, MINDY4, PIK3R3, RGS17, RPS6KA1, SH3RF1, SIPA1L3, SLC4A4, SND1, STRBP, TMPRSS4, USP4*
4/74	$2.498e^{-2}$	Organic Mental Disorders, Substance-Induced	*CDCP1, CSMD3, FAM184A, KLF12*
4/74	$2.498e^{-2}$	Absent reflex	*MFN2, NEB, TBCD, ZC4H2*

Table S5. Functional enrichment analysis of DPpGCs overrepresented in TS based on the GSEA annotation. Number of genes in the signal set 158. Number of genes in the background set 10771.

Count	*p*-value	GSEA	Genes
5/28	$2.285e^{-4}$	protein autoubiquitination	*LRRK2, RNF157, RNF213, UBE2D2, UBE2D3*
3/8	$3.282e^{-4}$	retrograde axonal transport	*AGBL4, DLG2, DST*
5/31	$3.769e^{-4}$	neuron projection cytoplasm	*AGBL4, AP3M1, DLG2, DST, LRRK2*
2/3	$7.986e^{-4}$	regulation of transmission of nerve impulse	*CHRNB4, FGF12*
2/3	$7.986e^{-4}$	cellular response to manganese ion	*BACE1, LRRK2*
4/22	$8.166e^{-4}$	axon cytoplasm	*AGBL4,,AP3M1, DLG2, DST*
8/95	$1.030e^{-3}$	cytoplasmic region	*AGBL4, AP3M1, DLG2, DNAH17, DNAH7, DST, LRRK2, WDR35*
5/39	$1.126e^{-3}$	single fertilization	*ADCY3, AR, SYT6, TRPC6, WDR48*
3/12	$1.230e^{-3}$	regulation of establishment of protein localization to mitochondrion	*LRRK2, NMT1, UBE2D3*
2/4	$1.585e^{-3}$	cellular response to testosterone stimulus	*AR, MSN*
2/4	$1.585e^{-3}$	intrinsic hand muscle atrophy	*FLNC, RYR1*
9/128	$1.966e^{-3}$	microtubule based movement	*ADCY3, AGBL4, AP3M1, CFAP44, DLG2, DNAH17, DNAH7, DST, WDR35*
6/63	$2.014e^{-3}$	sodium ion transmembrane transport	*ASIC2, ATP1A3, FGF12, HECW1, SLC17A7, SLC6A11*
2/5	$2.621e^{-3}$	seminiferous tubule development	*AR, WDR48*
2/5	$2.621e^{-3}$	megaloblastic anemia	*LMBRD1, PHGDH*
2/5	$2.621e^{-3}$	choroid plexus cyst	*PHGDH, ZSWIM6*
3/16	$2.985e^{-3}$	phototransduction	*ABCA4, ASIC2, NMT1*
3/16	$2.985e^{-3}$	anterograde axonal transport	*AGBL4, AP3M1, DLG2*
3/16	$2.985e^{-3}$	upper limb amyotrophy	*CYP7B1, FLNC, RYR1*
3/16	$2.985e^{-3}$	flushing	*ATP1A3, RUNX1, RYR1*
4/31	$3.090e^{-3}$	gait imbalance	*ATXN10, FLNC, LRRK2, SPTLC2*
6/69	$3.213e^{-3}$	neurotransmitter transport	*BACE1, CHRNB4, LRRK2, SLC17A7, SLC6A11, SYN2*
4/32	$3.481e^{-3}$	lysosomal transport	*AP3M1, DENND3, LRRK2, RNF157*
3/17	$3.581e^{-3}$	central nervous system neuron axonogenesis	*ARHGAP35, EPHB1, PLXNA4*
3/17	$3.581e^{-3}$	synaptic vesicle localization	*AP3M1, LRRK2, SYN2*
15/304	$3.826e^{-3}$	organelle assembly	*ARHGAP35, ATXN10, CFAP44, DNAH17, DNAH7, FLNC LRRK2, MSN, PTPRD, RNF213, STX18, TBC1D14, TPX2, WDR35, WWTR1*

Table S6. Functional enrichment analysis of DPpGCs overrepresented in TA based on the GSEA annotation. Number of genes in the signal set 155. Number of genes in the background set 10771.

Count	*p*-value	GSEA	Genes
2/2	$1.369e^{-4}$	regulation of growth rate	*RFTN1, TMPRSS4*
2/4	$8.124e^{-4}$	negative regulation of calcium ion dependent exocytosis	*GNAI2, REST*
2/5	$1.346e^{-3}$	fucose catabolic process	*FUT10, FUT8*
3/17	$1.369e^{-3}$	membrane protein ectodomain proteolysis	*ADAM10, LRIG2, SNX9*
13/317	$1.858e^{-3}$	metal ion transport	*CLCA1, GNAI2, HECW2, ITPR2, JPH2, KCNH5, NKAIN3, SLC25A37, SLC48A1, SLC4A4, THADA, TMEM163, TRPV1*
3/19	$1.919e^{-3}$	death in childhood	*ABCB11, JPH2, NEB*
9/179	$2.034e^{-3}$	infantile onset	*ABCB11, CA12, HYDIN, MFN2, NEB, PGAP3, REST, TBCD, VPS45*
3/20	$2.238e^{-3}$	phosphatidylinositol 3 phosphate binding	*JPH2, SNX24, ZFYVE28*
4/45	$3.690e^{-3}$	integrin mediated signaling pathway	*ADAM10, CDH17, FUT8, VAV3*
4/45	$3.690e^{-3}$	regulation of dendrite development	*CAPRIN1, COBL, CSMD3, HECW2*
2/8	$3.707e^{-3}$	regulation of membrane protein ectodomain proteolysis	*LRIG2, SNX9*
2/8	$3.707e^{-3}$	calcium release channel activity	*ITPR2, TRPV1*
2/8	$3.707e^{-3}$	duplication of the distal phalanx of hand	*PAH, ROR2*
6/99	$3.775e^{-3}$	phosphatidylinositol binding	*ITPR2, JPH2, SNX24, SNX9, TRPV1, ZFYVE28*
6/100	$3.969e^{-3}$	homophilic cell adhesion via plasma membrane adhesion molecules	*CDH1, CDH17, PCDHA1, PCDHA2, PCDHA3, PCDHA4*
7/133	$4.345e^{-3}$	cadherin binding	*CDH1, CDH17, DIAPH3, DOCK9, PPFIBP1, SND1, SNX9*
2/9	$4.738e^{-3}$	neuronal stem cell population maintenance	*FUT10, REST*
2/9	$4.738e^{-3}$	phosphatidylinositol 5 phosphate binding	*JPH2, SNX24*
9/210	$5.851e^{-3}$	regulation of proteolysis	*LRIG2, PSME4, REST, RPS6KA1, SH3RF1, SH3RF3, SIMC1, SNX9, TNIP1*
3/28	$6.006e^{-3}$	adherens junction organization	*ADAM10, CDH1, TBCD*
13/370	$6.808e^{-3}$	vesicle membrane	*ABCB11, ADAM10, ATAD3B, CLCA1, ITPR2, MCTP1, ROR2, SLC48A1, SNX24, SNX9, TMEM163, VPS45, ZFYVE28*
2/11	$7.157e^{-3}$	bile acid transmembrane transporter activity	*ABCB11, SLCO1C1*
2/11	$7.157e^{-3}$	abnormal tricuspid valve morphology	*JPH2, ROR2*

Table S7. CPpGCs for the sedentary and active SkM (TS and TA) and their function in SkM.

Gene	Function
ASB5<br>ankyrin repeat and SOCS box containing 5	Satellite cell-specific, expressed in both quiescent and activated satellite cells (Seale *et al.*, 2004).
LTBP1<br>latent transforming growth factor beta binding protein 1	Involved in the sequestration of latent TGF-β in the extracellular matrix (ECM) and the regulation of its activation in the extracellular environment (Todorovic & Rifkin, 2012).
PKCE<br>protein kinase C epsilon	Roles at sarcomeres and mitochondria in cardiomyocytes (Scruggs *et al.*, 2014).
PRDM16<br>PR/SET domain 16	Controls a brown fat/SkM switch (Seale *et al.*, 2008).
SYNC<br>syncoilin, intermediate filament protein	Highly expressed in skeletal and cardiac muscle; protein with structural role in striated muscle (Poon *et al.*, 2002).
Y_RNA<br>small non-coding RNA	Myoblasts produce small extracellular vesicles (ELV-MB) that contain Y_RNA (Rome *et al.*, 2019).
**...**	**Upper row genes are common CPpGCs in TS and TA; Lower row other CPpGCs of interest in alphabetic order**
NRXN1<br>neurexin 1	Required for efficient neurotransmission; Mutations in the gene implicated in nicotine dependence (Ching *et al.*, 2010).
PPP2R2C<br>protein phosphatase 2 regulatory subunit Bgamma	Implicated in the negative control of cell growth and division; downregulated in Alzheimer's disease brains (Leong *et al.*, 2020).
PRKCZ<br>protein kinase C zeta	Involved in proliferation, differentiation and secretion cellular processes; regulatory component of the insulin-like growth factor 1 receptor *IGF1R* (Seto *et al.*, 2015).
VSNL1<br>visinin like 1	Neuronal Ca^{2+} sensor protein, tumor suppressor by inhibiting cell proliferation, adhesion, and invasiveness; promoter of axonal differentiation and plasticity (Brackmann *et al.*, 2005).

References

1. Amoasii, L.; Holland W.; Sanchez-Ortiz, E.; Baskin, K.K.; Pearson, M.; Burgess, S.C.; Nelson, B.R.; Bassel-Duby, R.;Olson, E.N. A MED13-dependent skeletal muscle gene program controls systemic glucose homeostasis and hepatic metabolism. *Genes Dev*. **2016**, *30*, 434–446.
2. Bégin, S.; Guénard, F.; Tchernof, A.; Deshaies, Y.; Pérusse, L.; Biron, S.; Lescelleur, O.; Biertho, L.; Marceau, S.; Vohl,M.-C. Impact of NMT1 gene polymorphisms on features of the metabolic syndrome among severely obese patients. *Obes. Res. Open J*. **2015**, *2*, 101–110.
3. Bell, M.B.; Bush, Z.; McGinnis, G.R.; Rowe, G.C. Adult skeletal muscle deletion of Mitofusin 1 and 2 impedes exercise performance and training capacity. *J. Appl. Physiol*. (1985). **2019**, *126*, 341–353.
4. Bharti, S.K.; Khan, I.; Banerjee, T.; Sommers, J.A.;Wu, Y.; Brosh, R.M. Jr. Molecular functions and cellular roles of the ChlR1 (DDX11) helicase defective in the rare cohesinopathy Warsaw breakage syndrome. *Cell Mol. Life Sci*. **2014**, *71*,2625–2639.
5. Brackmann, M.; Schuchmann, S.; Anand, R.; Braunewell, K.H. Neuronal Ca2+ sensor protein VILIP-1 affects cGMP signalling of guanylyl cyclase B by regulating clathrin-dependent receptor recycling in hippocampal neurons. *J. Cell Sci*. **2005**, *118*, 2495–2505.
6. Chen, M.; Zhang, W.; Lu, X.; Hoggatt, A.M.; Gunst, S.J.; Kassab, G.S.; Tune, J.D.; Herring, B.P. Regulation of 130-kDa Smooth Muscle Myosin Light Chain Kinase Expression by an Intronic CArG Element. *J. Biol. Chem.* **2013**, *288*, 34647–3457.
7. Ching, M.S.; Shen, Y.; Tan, W.H.; Jeste, S.S.; Morrow, E.M.; Chen, X.; Mukaddes, N.M.; Yoo, S.Y.; Hanson, E.; Hundley, R.; et al. Children's Hospital Boston Genotype Phenotype Study Group. Deletions of NRXN1 (neurexin-1) predispose to a wide spectrum of developmental disorders. *Am. J. Med. Genet B Neuropsychiatr. Genet.* **2010**, *153B*, 937–947.
8. Choi, W.I.; Yoon, J.H.; Choi, S.H.; Jeon, B.N.; Kim, H.; Hur, M.W. Proto-oncoprotein Zbtb7c and SIRT1 repression: implications in high-fat diet-induced and age-dependent obesity. *Exp. Mol. Med.* **2021**, *53*, 917–932.

9. Cheret, C.; Willem, M.; Fricker, F.R.; Wende, H.; Wulf-Goldenberg, A.; Tahirovic, S.; Nave, K.A.; Saftig, P.; Haass, C.; Garratt, A.N.; Bennett, D.L.; et al. Bace1 and Neuregulin-1 cooperate to control formation and maintenance of muscle spindles. *EMBO J.* **2013**, *32*, 2015–2028.
10. Faralli, H.; Martin, E.; Coré, N.; Liu, Q.C.; Filippi, P.; Dilworth, F.J.; Caubit, X.; Fasano, L. Teashirt-3, a novel regulator of muscle differentiation, associates with BRG1-associated factor 57 (BAF57) to inhibit myogenin gene expression. *J. Biol. Chem*. **2011**, *286*, 23498–23510.
11. Grarup, N.; Moltke, I.; Andersen, M.K.; Dalby, M.; Vitting-Seerup, K.; Kern, T.; Mahendran, Y.; Jørsboe, E.; Larsen, C.V.L.; Dahl-Petersen, I.K.; et al. Loss-of-function variants in ADCY3 increase risk of obesity and type 2 diabetes. *Nat. Genet*. **2018**, *50*, 172–174.
12. Joshi, A.S.; Ragusa, J.V.; Prinz, W.A.; Cohen, S. Multiple C2 domain-containing transmembrane proteins promote lipid droplet biogenesis and growth at specialized endoplasmic reticulum subdomains. *Mol. Biol. Cell.* **2021**, *32*, 1147–1157.
13. Jung, T.W.; Park, J.; Sun, J.L.; Ahn, S.H.; Abd El-Aty, A.M.; Hacimuftuoglu, A.; Kim, H.C.; Shim, J.H.; Shin, S.; Jeong, J.H. Administration of kynurenic acid reduces hyperlipidemia-induced inflammation and insulin resistance in skeletal muscle and adipocytes. *Mol. Cell Endocrinol*. **2020**, *518*, 110928.
14. Kamizaki, K.; Endo, M.; Minami, Y.; Kobayashi, Y. Role of noncanonical Wnt ligands and Ror-family receptor tyrosine kinases in the development, regeneration, and diseases of the musculoskeletal system. Dev. Dyn. 2021, 250, 27-38.
15. Kim, J.; Kim, J.; Min, H.; Oh, S.; Kim, Y.; Lee, A.H.; Park, T. Joint identification of genetic variants for physical activity in Korean population. *Int. J. Mol. Sci*. **2014**, *15*, 12407–12421.
16. Kotani, Y.; Morito, D.; Yamazaki, S.; Ogino, K.; Kawakami, K.; Takashima, S.; Hirata, H.; Nagata, K. Neuromuscular regulation in zebrafish by a large AAA+ ATPase/ubiquitin ligase, mysterin/RNF213. *Sci. Rep*. **2015**, *5*, 16161.
17. Leong, W.; Xu, W.; Wang, B.; Gao, S.; Zhai, X.; Wang, C.; Gilson, E.; Ye, J.; Lu, Y. PP2A subunit PPP2R2C is downregulated in the brains of Alzheimer's transgenic mice. *Aging (Albany NY).* **2020**, *12*, 6880–-6890.
18. Li, C.; Mo, D.; Li, M.; Zheng, Y.; Li, Q.; Ou, S.; Zhang, Z. Age-related but not longevity-related genes are found by weighted gene co-expression network analysis in the peripheral blood cells of humans. *Genes Genet. Syst*. **2019**, *93*, 221–228.
19. Mizuno, S.; Yoda, M.; Shimoda, M.; Tohmonda, T.; Okada, Y.; Toyama, Y.; Takeda, S.; Nakamura, M.; Matsumoto, M.; Horiuchi, K. A Disintegrin and Metalloprotease 10 (ADAM10) Is Indispensable for Maintenance of the Muscle Satellite Cell *Pool. J. Biol. Chem*. **2015**, *290*, 28456–28464.
20. Ndiaye, F.K.; Huyvaert, M.; Ortalli, A.; Canouil, M.; Lecoeur, C.; Verbanck, M.; Lobbens, S.; Khamis, A.; Marselli, L.; Marchetti, P.; et al. The expression of genes in top obesity-associated loci is enriched in insula and substantia nigra brain regions involved in addiction and reward. *Int. J. Obes (Lond).* **2020**, *44*, 539–543.
21. Palmer, C.J.; Bruckner, R.J.; Paulo, J.A.; Kazak, L.; Long, J.Z.; Mina, A.I.; Deng, Z.; LeClair, K.B.; Hall, J.A.; Hong, S.; et al. Cdkal1, a type 2 diabetes susceptibility gene, regulates mitochondrial function in adipose tissue. *Mol. Metab*. **2017**, *6*, 1212–1225.
22. Pan, H.; Xu, X.; Wu, D.; Qiu, Q.; Zhou, S.; He, X.; Zhou, Y.; Qu, P.; Hou, J.; He, J.; et al. Novel somatic mutations identified by whole-exome sequencing in muscle-invasive transitional cell carcinoma of the bladder. *Oncol. Lett*. **2016**, *11*, 1486-1492.
23. Peres de Oliveira, A.; Kazuo Issayama, L.; Betim Pavan, I.C.; Riback Silva, F.; Diniz Melo-Hanchuk, T.; Moreira Simabuco, F.; Kobarg, J. Checking NEKs: Overcoming a Bottleneck in Human Diseases. *Molecules.* **2020**, *25*, 1778.
24. Polge, C.; Attaix, D.; Taillandier, D. Role of E2-Ub-conjugating enzymes during skeletal muscle atrophy. *Front. Physiol*. **2015**, *6*, 59.
25. Poon, E.; Howman, E.V.; Newey, S.E.; Davies, K.E. Association of syncoilin and desmin: linking intermediate filament proteins to the dystrophin-associated protein complex. *J. Biol. Chem.* **2002**, *277*, 3433–3439. 1226.
26. Radom-Aizik, S.; Zaldivar, F. Jr.; Leu, S.Y.; Cooper, D.M. Brief bout of exercise alters gene expression in peripheral blood mononuclear cells of early- and late-pubertal males. *Pediatr. Res*. **2009**, *65*, 447−452.
27. Rogowski, K.; van Dijk, J.; Magiera, M.M.; Bosc, C.; Deloulme, J.C.; Bosson, A.; Peris, L.; Gold, N.D.; Lacroix, B.; Bosch Grau, M.; et al. A Family of Protein-Deglutamylating Enzymes Associated with Neurodegeneration. *Cell*. **2010**, *143*, 564–578.
28. Rome, S.; Forterre, A.; Mizgier, M.L.; Bouzakri, K. Skeletal Muscle-Released Extracellular Vesicles: State of the Art. *Front. Physiol*. **2019**, *10*, 929.
29. Rusu, V.; Hoch, E.; Mercader, J.M.; Tenen, D.E.; Gymrek, M.; Hartigan, C.R.; DeRan, M.; von Grotthuss, M.; Fontanillas, P.; Spooner, A.; et al. Type 2 Diabetes Variants Disrupt Function of SLC16A11 through Two Distinct Mechanisms. *Cell*. **2017**, *170*, 199–212.e20.
30. Sarkar, P.; Thirumurugan, K. New insights into TNFα/PTP1B and PPARγ pathway through RNF213- a link between inflammation, obesity, insulin resistance, and Moyamoya disease. *Gene*. **2021**, *771*, 145340.
31. Scruggs, S.B.; Wang, D.; Ping, P. PRKCE gene encoding protein kinase C-epsilon-Dual roles at sarcomeres and mitochondria in cardiomyocytes. *Gene*. **2016**, *590*, 90–96.
32. Seale, P.; Bjork, B.; Yang, W.; Kajimura, S.; Chin, S.; Kuang, S.; Scimè, A.; Devarakonda, S.; Conroe, H.M.; Erdjument-Bromage, H.; et al. PRDM16 controls a brown fat/skeletal muscle switch. *Nature*. **2008**, *454*, 961–967.
33. Selman, C.; Tullet, J.M.; Wieser, D.; Irvine, E.; Lingard, S.J.; Choudhury, A.I.; Claret, M.; Al-Qassab, H.; Carmignac, D.; Ramadani, F.; et al. Ribosomal protein S6 kinase 1 signaling regulates mammalian life span. *Science*. **2009**, *326*, 140–144.
34. Setiawan, I.; Sanjaya, A.; Lesmana, R.; Yen, P.M.; Goenawan, H. Hippo pathway effectors YAP and TAZ and their association with skeletal muscle ageing. *J. Physiol. Biochem.* **2021**, *77*, 63–73.

35. Seto, K.K.; Andrulis, I.L. Atypical protein kinase C zeta: potential player in cell survival and cell migration of ovarian cancer. *PLoS One*. **2015**, *10*, e0123528.
36. Shamilov, R.; Aneskievich, B.J. TNIP1 in Autoimmune Diseases: Regulation of Toll-like Receptor Signaling. *J. Immunol. Res*. **2018**, *2018*, 3491269.
37. Söhle, J.; Machuy, N.; Smailbegovic, E.; Holtzmann, U.; Grönniger, E.; Wenck, H.; Stäb, F.; Winnefeld, M. Identification of new genes involved in human adipogenesis and fat storage. *PLoS One*. **2012**, *7*, e31193.
38. Sun, C.; De Mello, V.; Mohamed, A.; Ortuste Quiroga, H.P.; Garcia-Munoz, A.; Al Bloshi, A.; Tremblay, A.M.; von Kriegsheim, A.; Collie-Duguid, E.; Vargesson, N.; et al. Common and Distinctive Functions of the Hippo Effectors Taz and Yap in Skeletal Muscle Stem Cell Function. *Stem Cells.* **2017**, *35*, 1958–1972.
39. Thiel, G. Synapsin I, synapsin II, and synaptophysin: marker proteins of synaptic vesicles. *Brain Pathol*. **1993**, *3*, 87–95.
40. Todorovic, V.; Rifkin, D.B. LTBPs, more than just an escort service. *J. Cell Biochem*. **2012**, *113*, 410–418.
41. Uezumi, A.; Nakatani, M.; Ikemoto-Uezumi, M.; Yamamoto, N.; Morita, M.; Yamaguchi, A.; Yamada, H.; Kasai, T.; Masuda, S.; Narita, A.; et al. Cell-Surface Protein Profiling Identifies Distinctive Markers of Progenitor Cells in Human Skeletal Muscle. *Stem. Cell Reports*. **2016**, *7*, 263–278.
42. Weng, X.; Lin, D.; Huang, J.T.J.; Stimson, R.H.; Wasserman, D.H.; Kang, L. Collagen 24 α1 Is Increased in Insulin-Resistant Skeletal Muscle and Adipose Tissue. *Int. J. Mol. Sci*. 2020, *21*, 5738.
43. Wiel, C.; Lallet-Daher, H.; Gitenay, D.; Gras, B.; Le Calvé, B.; Augert, A.; Ferrand, M.; Prevarskaya, N.; Simonnet, H.;Vindrieux, D.; et al. Endoplasmic reticulum calcium release through ITPR2 channels leads to mitochondrial calcium accumulation and senescence. *Nat. Commun*. **2014**, *5*, 3792.
44. Witt, C.C.; Burkart, C.; Labeit, D.; McNabb, M.; Wu, Y.; Granzier, H.; Labeit, S. Nebulin regulates thin filament length, contractility, and Z-disk structure in vivo. *EMBO J*. 2006, *25*, 3843–3855.
45. Zhao, Y.; Huang, G.; Chen, Z.; Fan, X.; Huang, T.; Liu, J.; Zhang, Q.; Shen, J.; Li, Z.; Shi, Y. Four Loci Are Associated with Cardiorespiratory Fitness and Endurance Performance in Young Chinese Females. *Sci. Rep*. **2020**, *10*, 10117.
46. Ziegler, D.V.; Vindrieux, D.; Goehrig, D.; Jaber, S.; Collin, G.; Griveau, A.; Wiel, C.; Bendridi, N.; Djebali, S.;Farfariello, V.; et al. Calcium channel ITPR2 and mitochondria-ER contacts promote cellular senescence and aging. *Nat. Commun*. **2021**, *12*, 720.
47. Zykovich, A.; Hubbard, A.; Flynn, J.M.; Tarnopolsky, M.; Fraga, M.F.; Kerksick, C.; Ogborn, D.; MacNeil, L.; Mooney, S.D.; Melov, S. Genome-wide DNA methylation changes with age in disease-free human skeletal muscle. *Aging Cell*. **2014**, *13*, 360–366.

Supplementary Figures

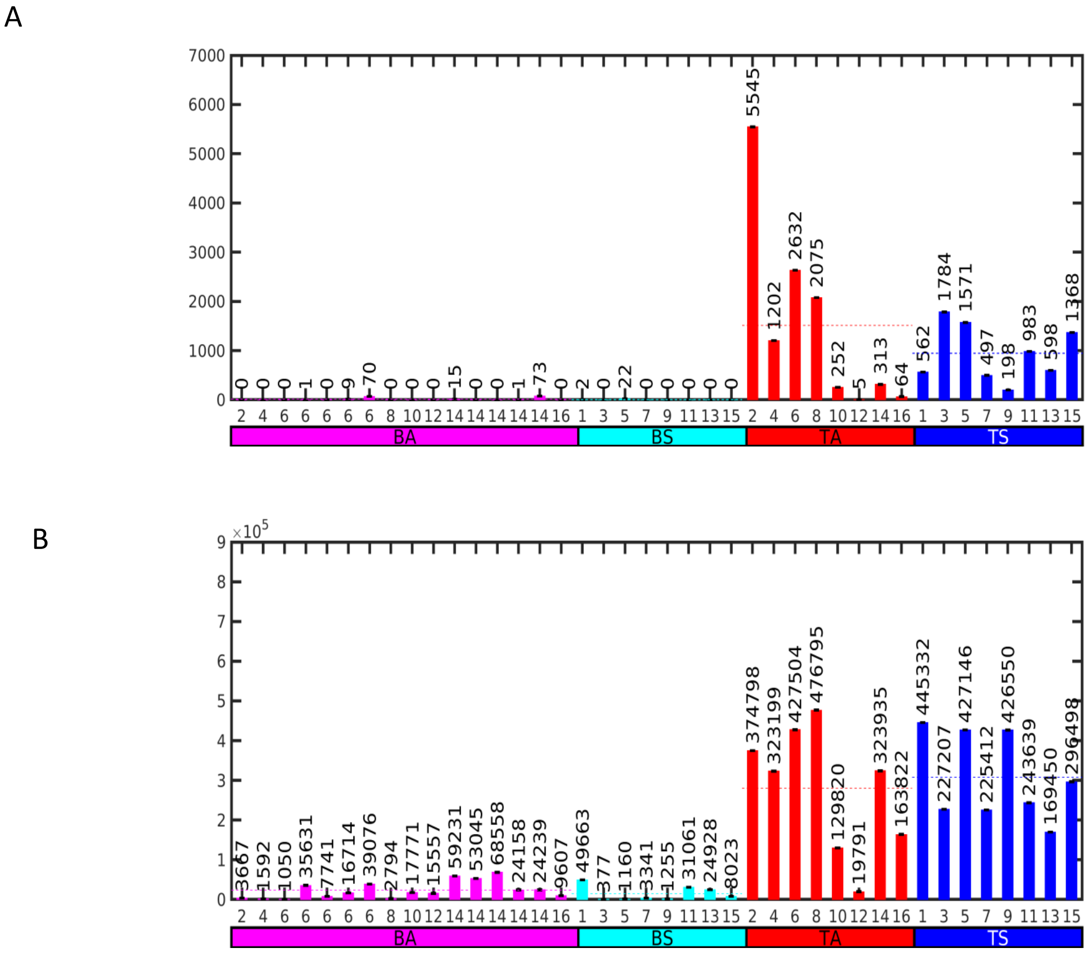

Figure S1. Mitochondrial eccDNA analysis. (A) Number of split reads per sample in mitochondrial eccDNA. (B) Number of split reads per sample in non-mitochondrial eccDNA. The horizontal dotted lines mark the positions of the mean values of each of the four groups (BA, BS, TA, TS).

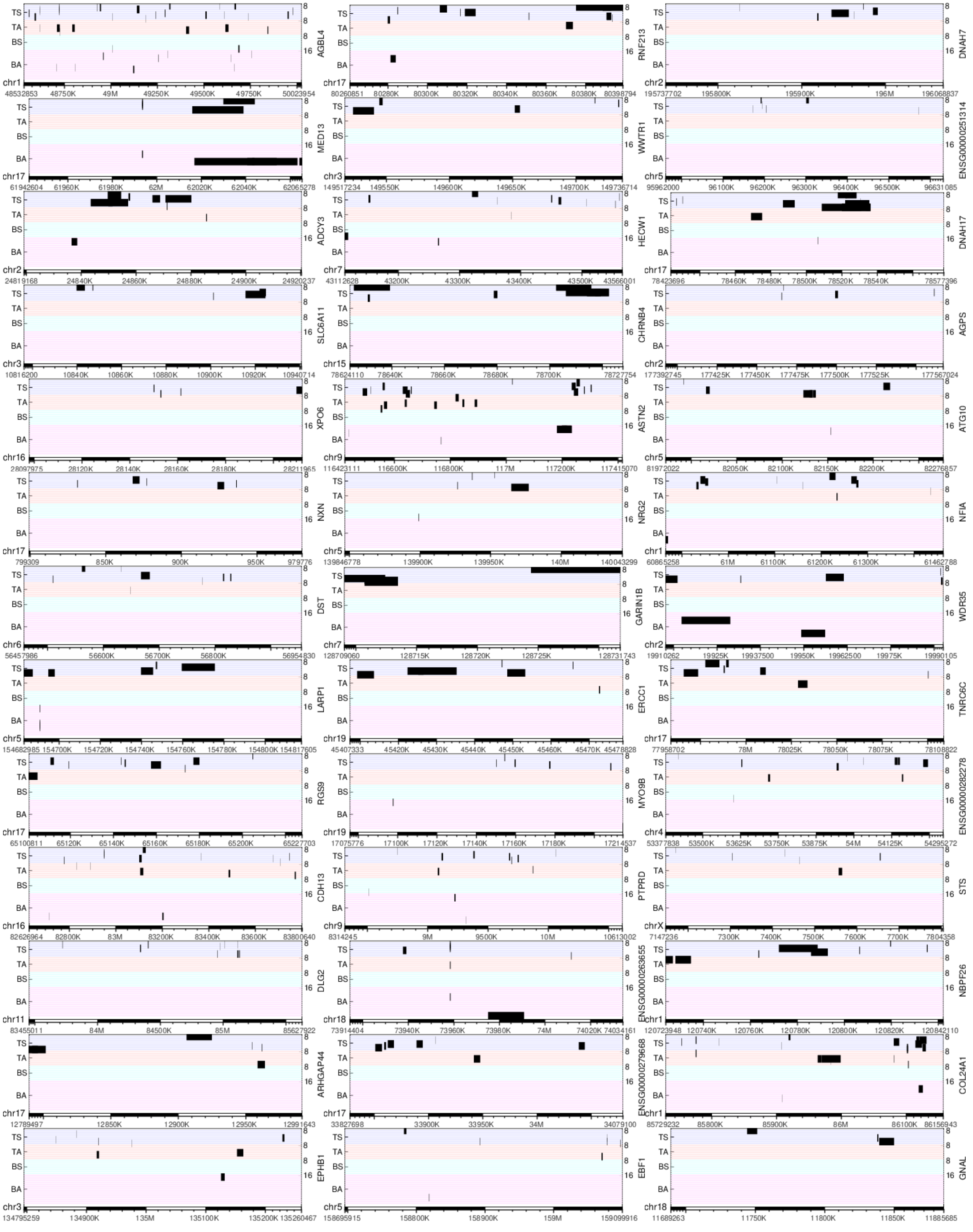

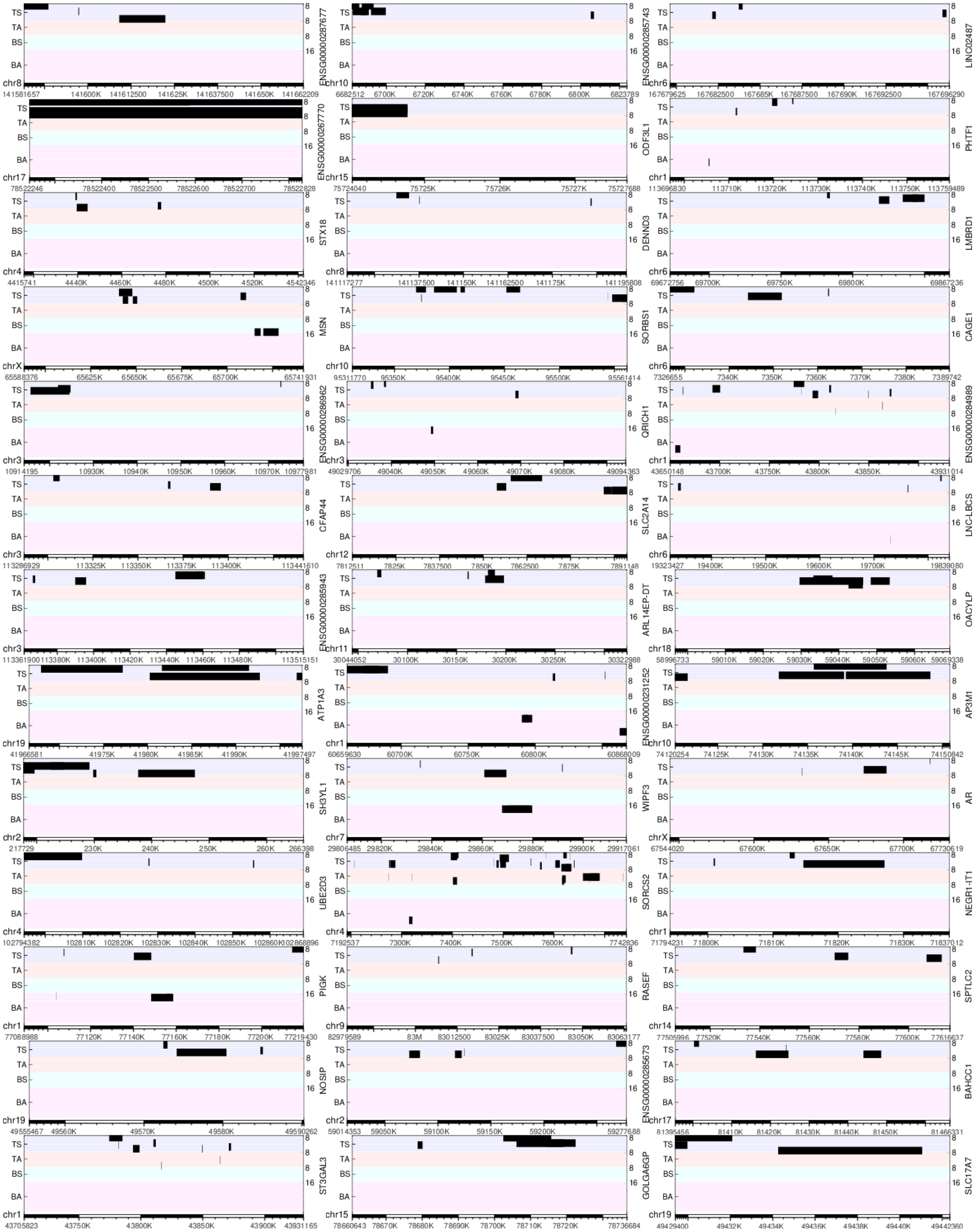
TS
TA
BS
BA
chr8
ENSG00000287677
chr10
ENSG00000285743
chr6
LINC02487
chr17
ENSG00000267770
chr15
ODF3L1
chr1
PHTF1
chr4
STX18
chr8
DENND3
chr6
LMBRD1
chrX
MSN
chr10
SORBS1
chr6
CAGE1
chr3
ENSG00000286962
chr3
QRICH1
chr1
ENSG00000284989
chr3
CFAP44
chr12
SLC2A14
chr6
LNC-LBCS
chr3
ENSG00000285943
chr11
ARL14EP-DT
chr18
OACYLP
chr19
ATP1A3
chr1
ENSG00000231252
chr10
AP3M1
chr2
SH3YL1
chr7
WIPF3
chrX
AR
chr4
UBE2D3
chr4
SORCS2
chr1
NEGR1-IT1
chr1
PIGK
chr9
RASEF
chr14
SPTLC2
chr19
NOSIP
chr2
ENSG00000285673
chr17
BAHCC1
chr1
ST3GAL3
chr15
GOLGA6GP
chr19
SLC17A7

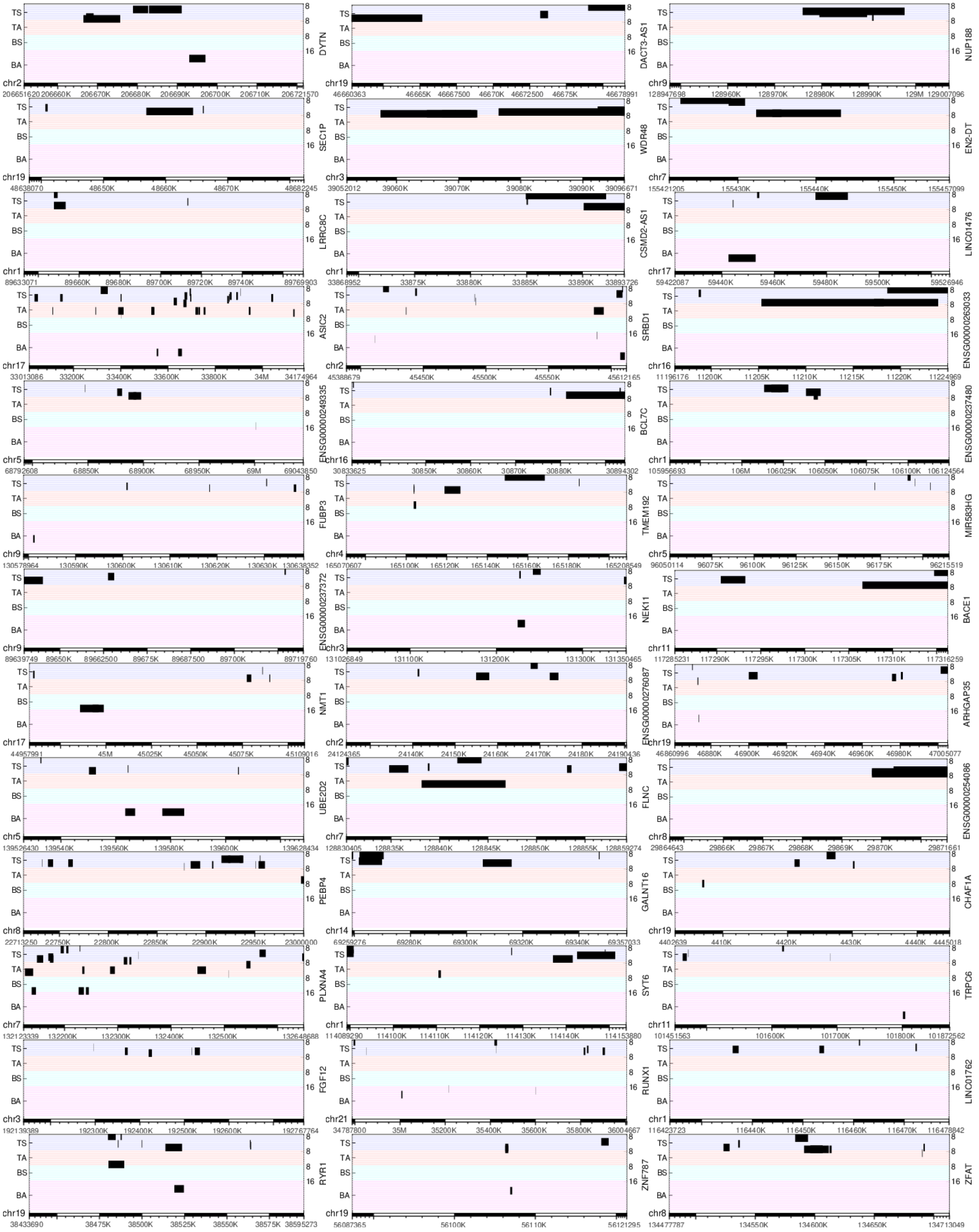

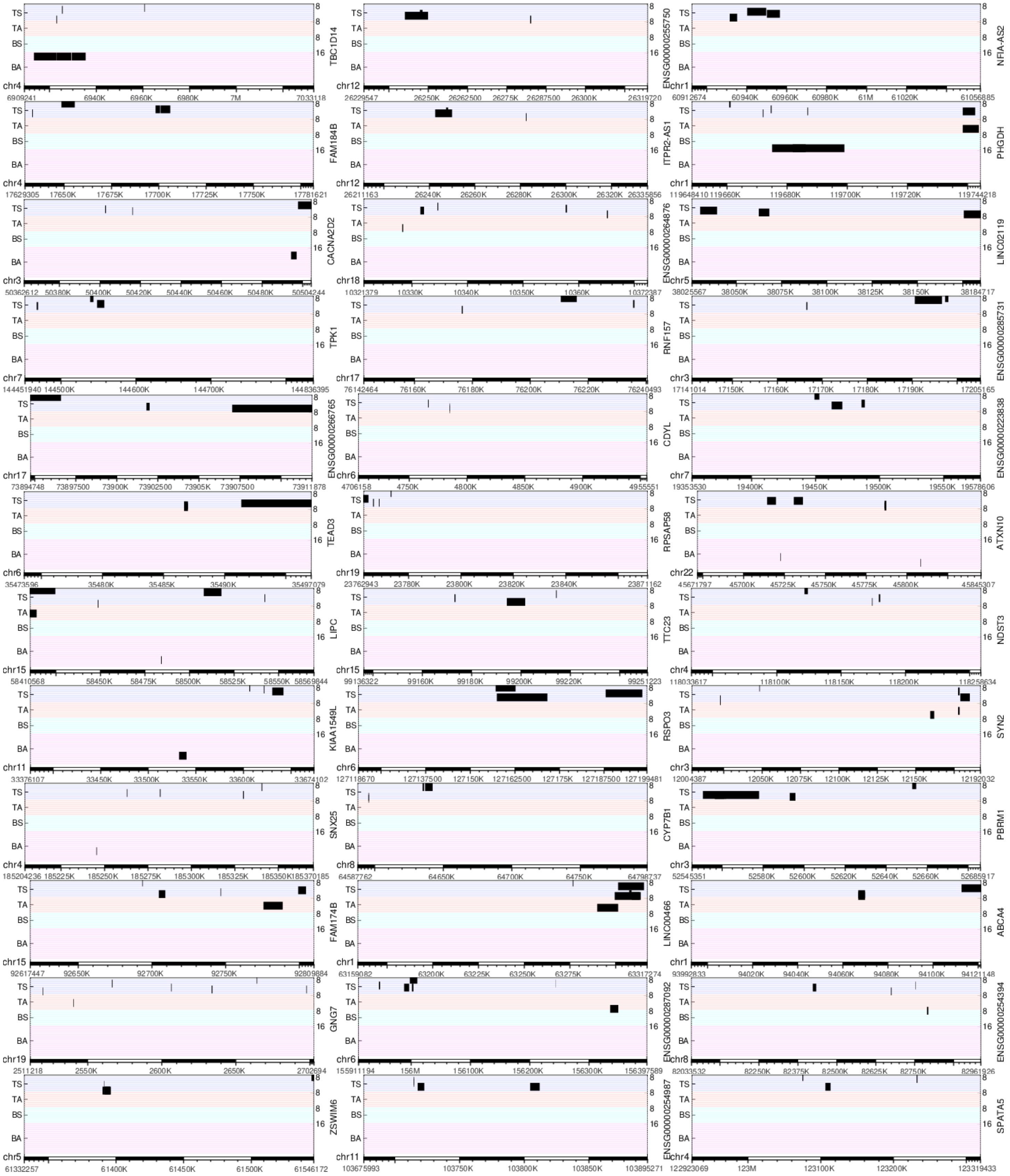
TBC1D14
ENSG00000255750
NFIA-AS2
FAM184B
ITPR2-AS1
PHGDH
CACNA2D2
ENSG00000264876
LINC02119
TPK1
RNF157
ENSG00000285731
ENSG00000266765
CDYL
ENSG00000223838
TEAD3
RPSAP58
ATXN10
LIPC
TTC23
NDST3
KIAA1549L
RSPO3
SYN2
SNX25
CYP7B1
PBRM1
FAM174B
LINC00466
ABCA4
GNG7
ENSG00000287092
ENSG00000254394
ZSWIM6
ENSG00000254987
SPATA5

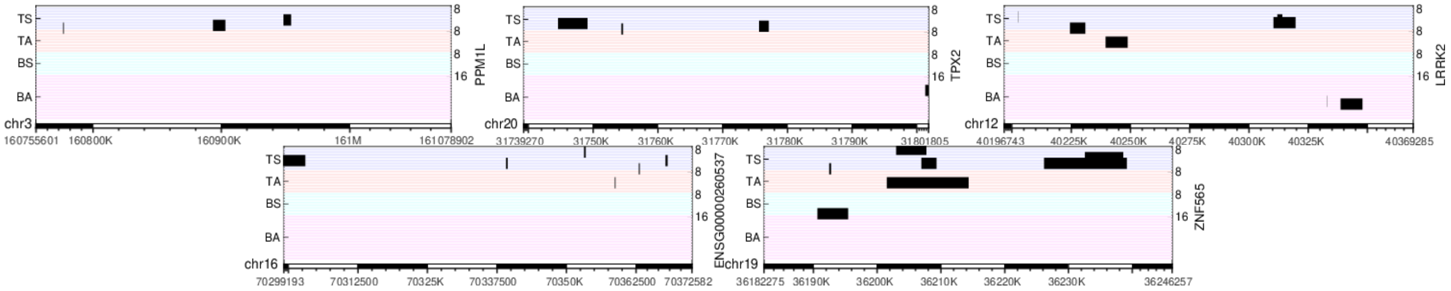

Figure S2. Track plots of the *loci* of the 158 top-ranked up-DPpGCs in TS in relation to TA. The statistically most significant up-DPpGC is *AGBL4* with *p*-value 0.0010762.

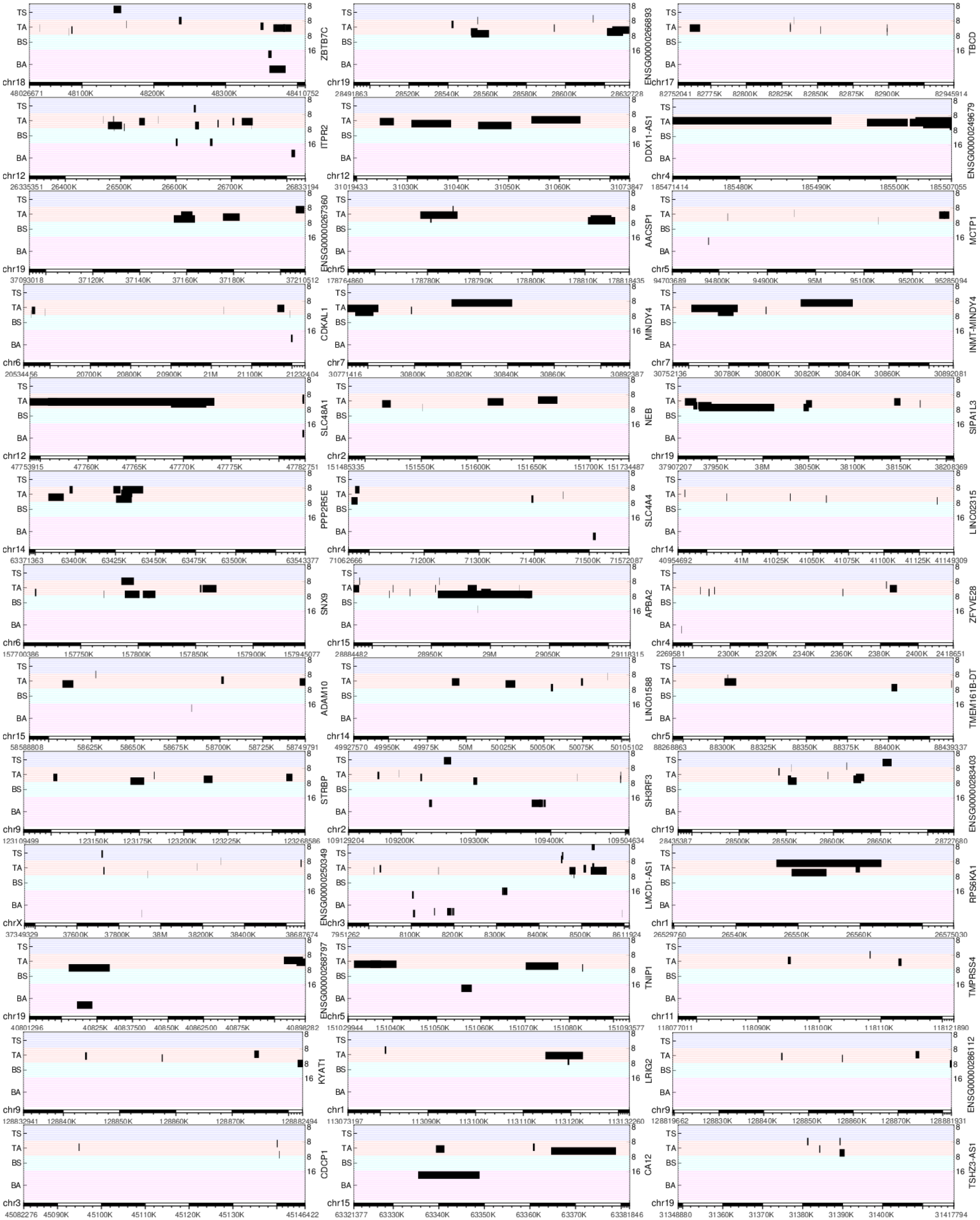
TS
TA
BS
BA
ZBTB7C
ENSG00000266893
TBCD
ITPR2
DDX11-AS1
ENSG00000249679
ENSG00000267360
AACSP1
MCTP1
CDKAL1
MINDY4
INMT-MINDY4
SLC48A1
NEB
SIPA1L3
PPP2R5E
SLC4A4
LINC02315
SNX9
APBA2
ZFYVE28
ADAM10
LINC01588
TMEM161B-DT
STRBP
SH3RF3
ENSG00000283403
ENSG00000250349
LMCD1-AS1
RPS6KA1
ENSG00000268797
TNIP1
TMPRSS4
KYAT1
LRIG2
ENSG00000286112
CDCP1
CA12
TSHZ3-AS1

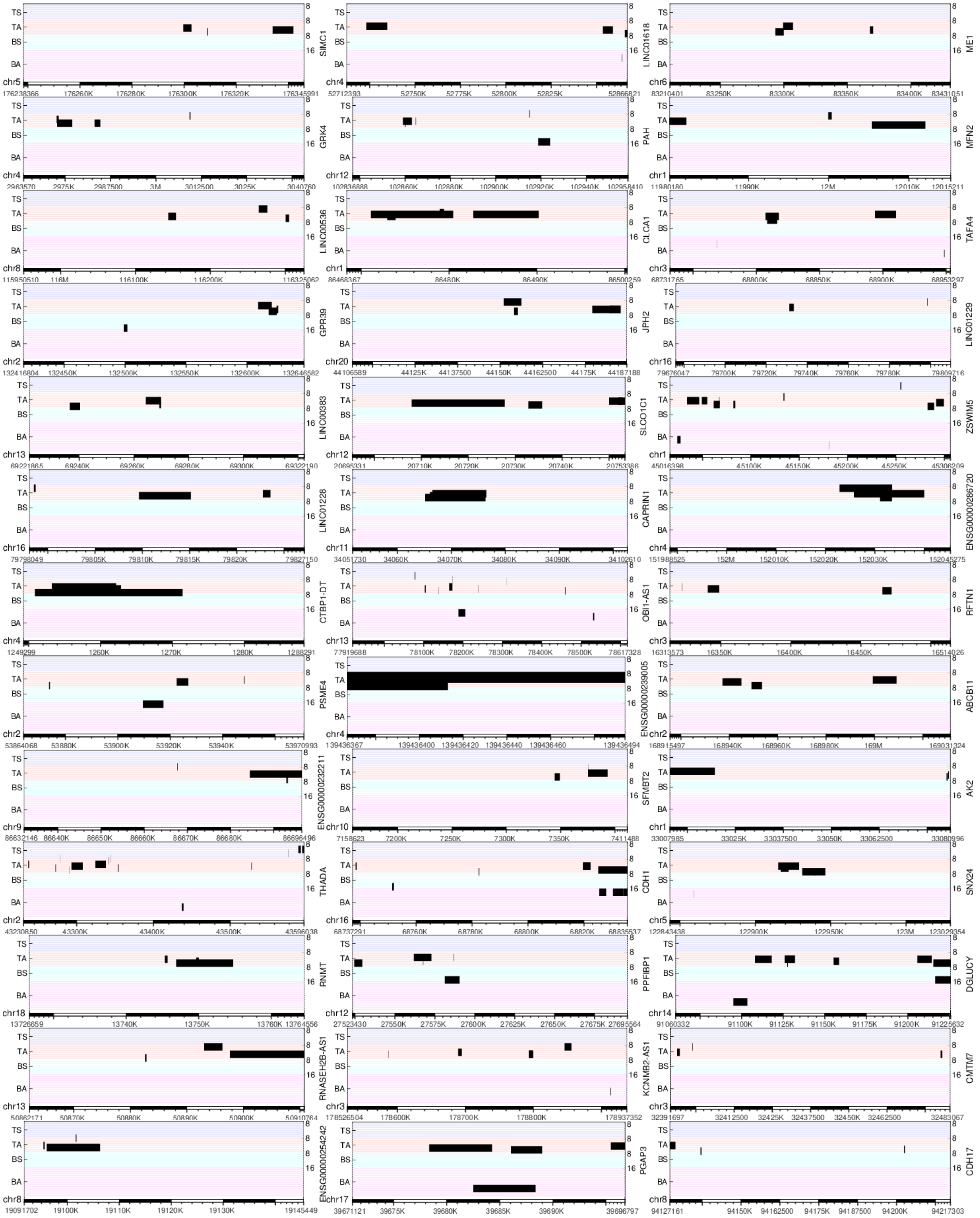

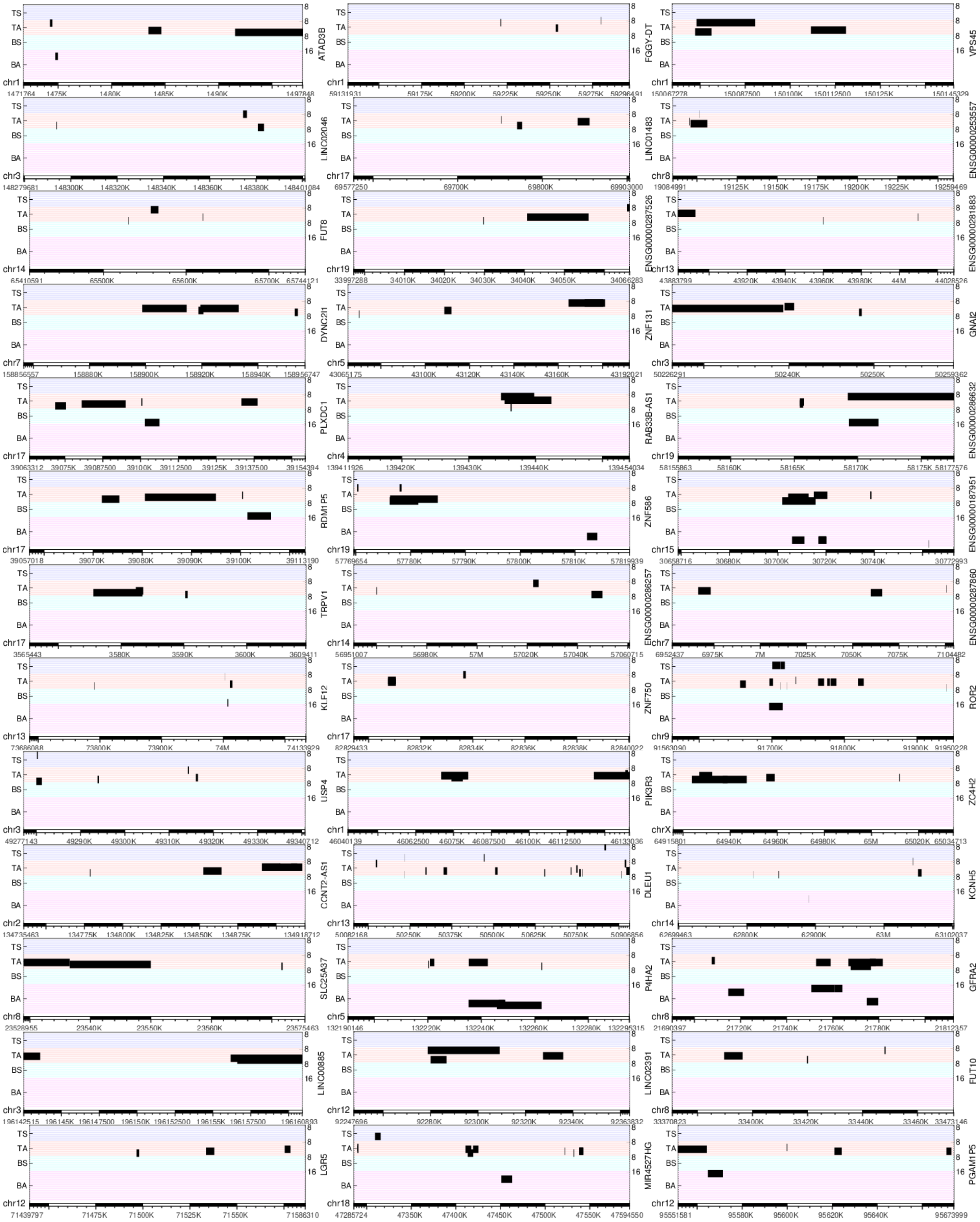
TS
TA
BS
BA
ATAD3B
FGGY-DT
VPS45
LINC02046
LINC01483
ENSG00000253557
FUT8
ENSG00000287526
ENSG00000281883
DYNC2I1
ZNF131
GNAI2
PLXDC1
RAB33B-AS1
ENSG00000286632
RDM1P5
ZNF586
ENSG00000187951
TRPV1
ENSG00000286257
ENSG00000287860
KLF12
ZNF750
ROR2
USP4
PIK3R3
ZC4H2
CCNT2-AS1
DLEU1
KCNH5
SLC25A37
P4HA2
GFRA2
LINC00885
LINC02391
FUT10
LGR5
MIR4527HG
PGAM1P5
chr1
chr3
chr17
chr8
chr14
chr19
chr13
chr7
chr5
chr4
chr15
chr9
chrX
chr2
chr12
chr18

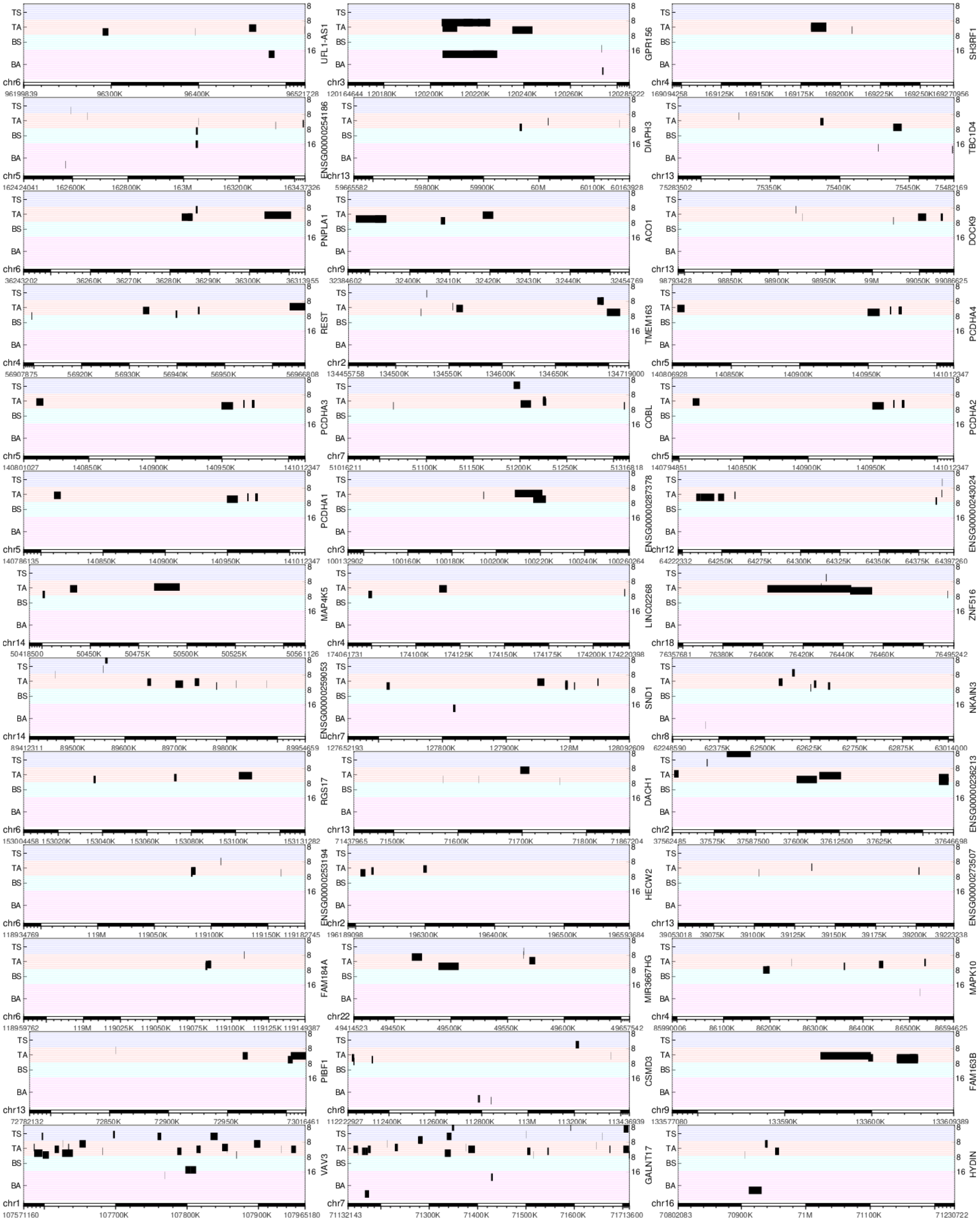

Figure S3. Track plots of the *loci* of the 156 top-ranked up-DPpGCs in TA in relation to TS. The statistically most significant differentially up-DPpGC in TA is *ZBTB7C* with *p*-value 0.0023656.

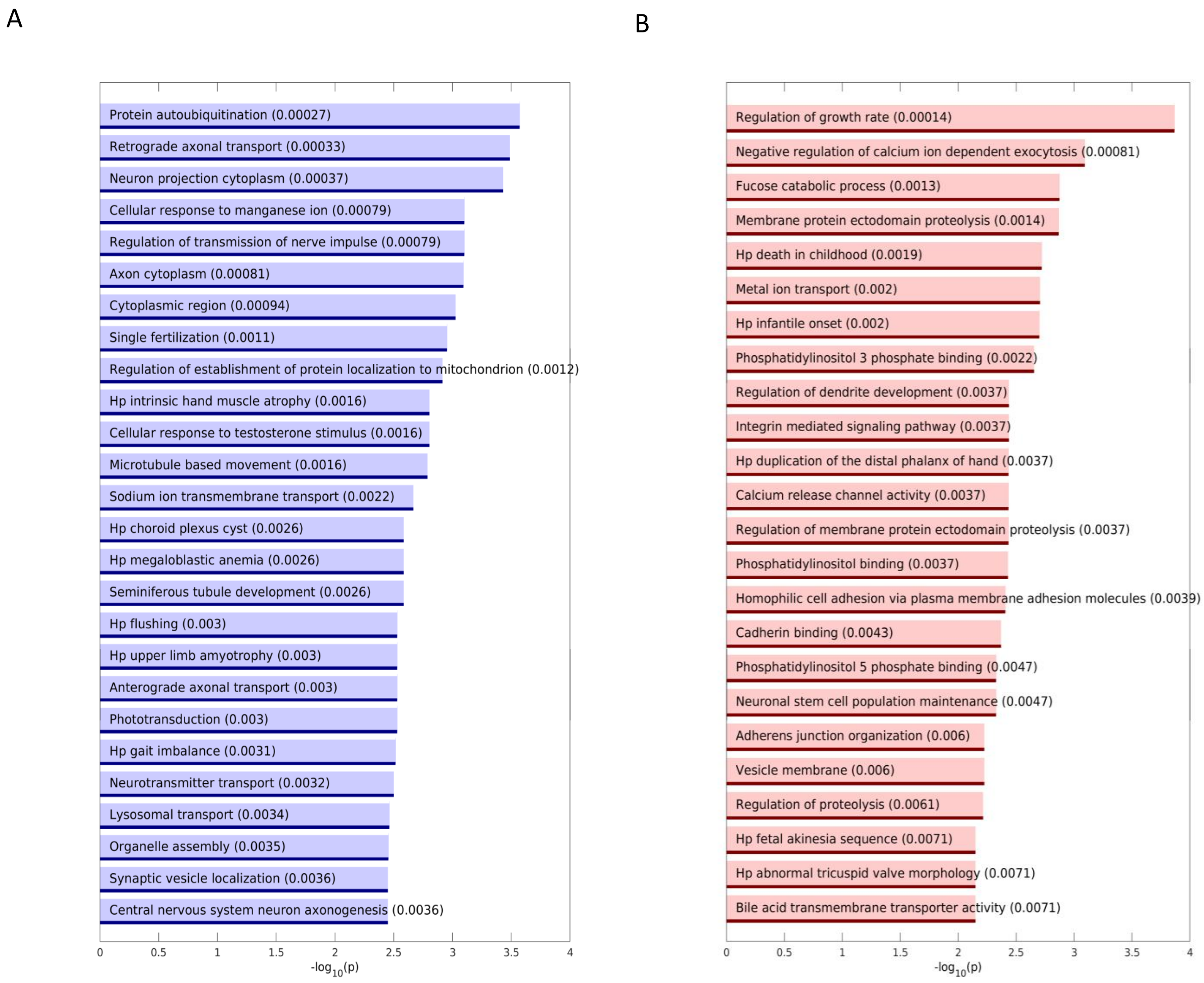

Figure S4. Bar plots of the –log$_{10}$(*p*-value) of the significantly enriched GSEA terms of up-DPpGCs. (A) TS. .(B) TA. Longer bars correspond to higher statistical significance of the enrichment (the *p*-values are given in parentheses).

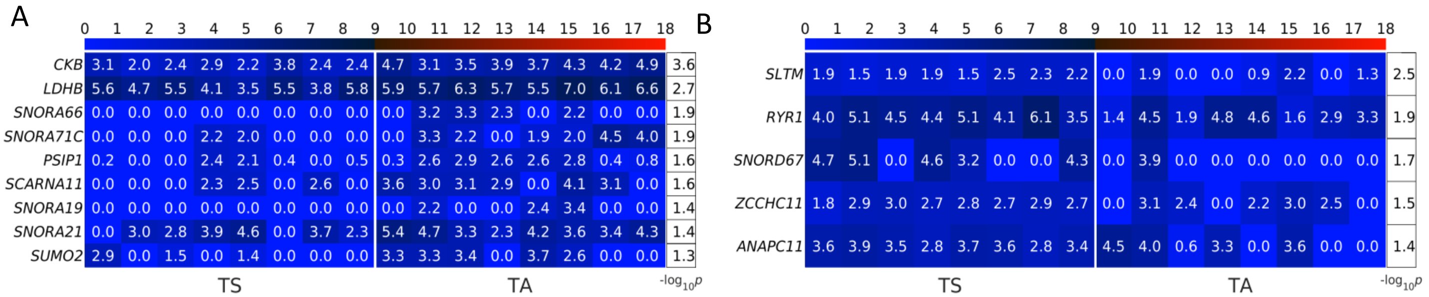

Figure S5. Transcriptomics analysis. Heatmaps of the transcriptomics level of DEGs up-regulated in (A) TA and (B) TS. The color bar codifies the Fragments per Kilobase of Exon per Million Mapped Fragments (FPKMs) in a $\log_{10}$ scale. Higher count corresponds to a redder color.

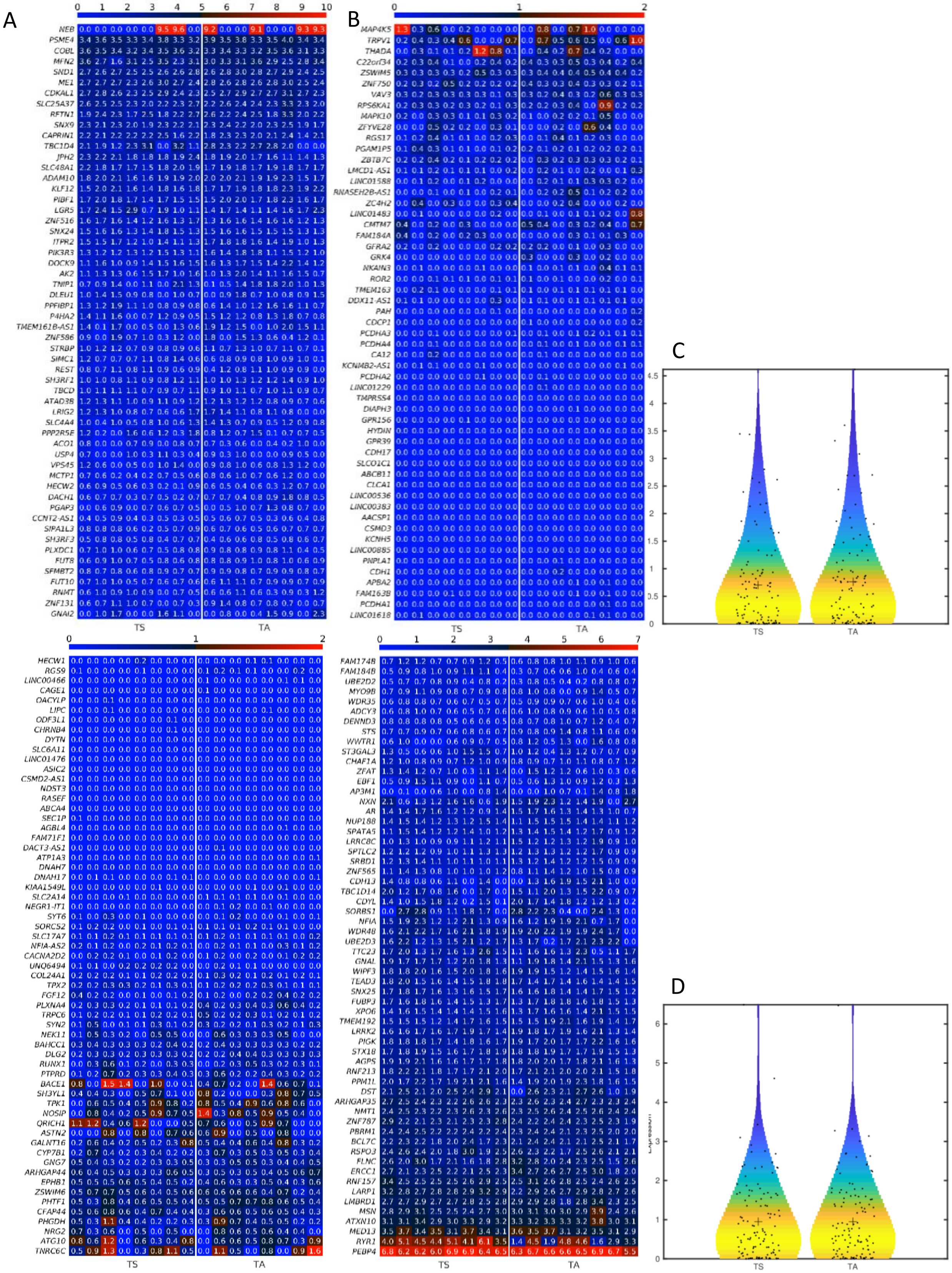

Figure S6. Transcriptomics analysis of the genes associated with DPpGCs. Heatmaps of the transcriptomics level of genes associated with DPpGCs in TS (A) and in TA (B). The color bar codifies the FPKMs in a log_{10} scale. Higher FPKMs correspond to a redder color. Violins plots associated to the expression distribution of the genes in TS (C) and in TA (D). The black crosses indicate the medians of the distributions. The black points represent the spread of the expression of the genes used to build the distributions.

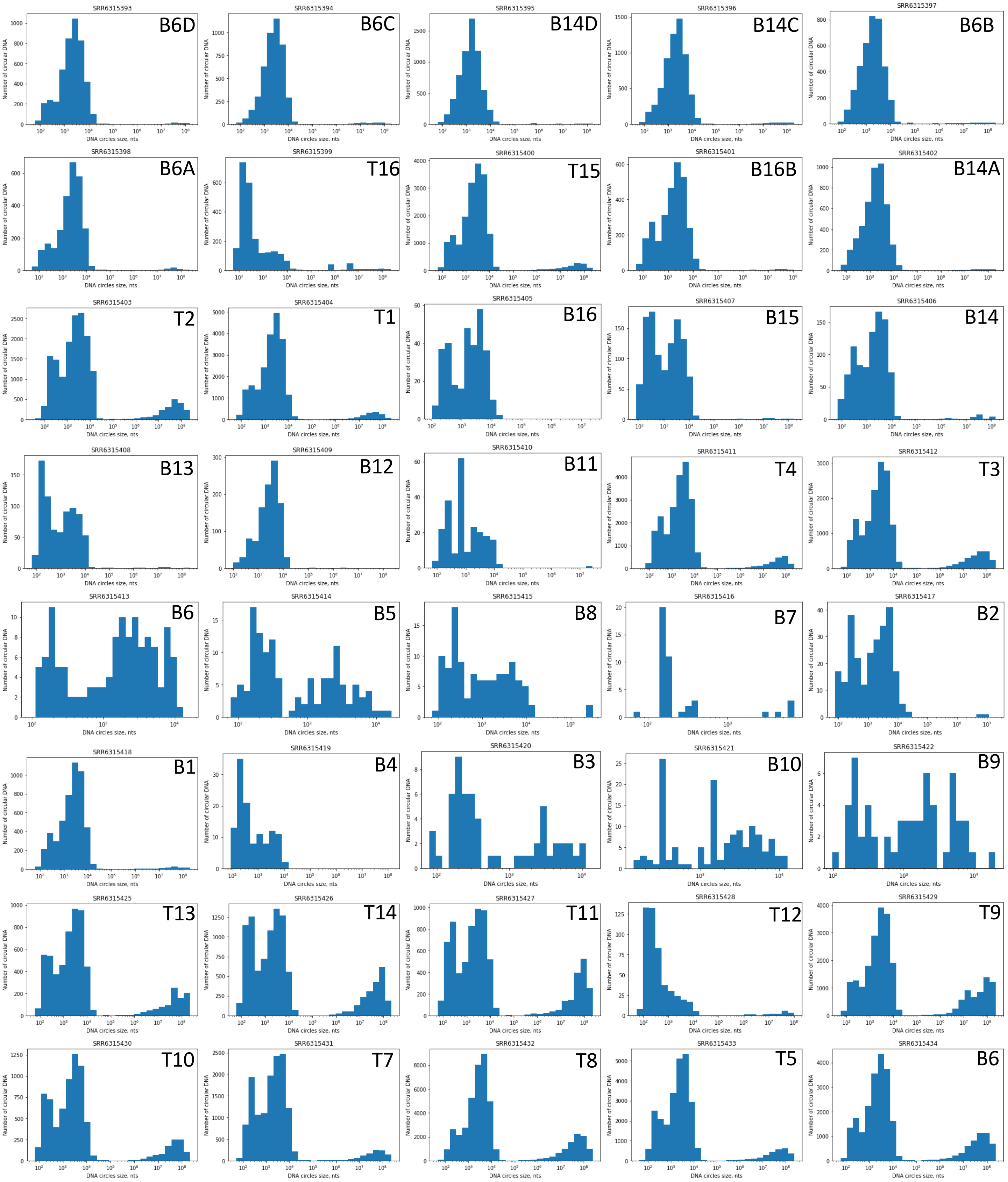

Figure S7. Histograms of the number of eccDNA mapped with Circle_finder (Kumar *et al.*, 2020) using as arguments the hg38 built of the genome and minNonOverlap between two split reads equal to 10. T, SkM tissue sample, B, blood (leukocytes) sample. Odd numbers correspond to sedentary samples; even numbers correspond to active lifestyle samples.
